# Ultraviolet Quantum Emitters in Hexagonal Boron Nitride from Carbon Clusters

**DOI:** 10.1021/acs.jpclett.2c00665

**Published:** 2022-04-01

**Authors:** Song Li, Anton Pershin, Gergő Thiering, Péter Udvarhelyi, Adam Gali

**Affiliations:** † Wigner Research Centre for Physics, P.O. Box 49, H-1525 Budapest, Hungary; ‡ Department of Atomic Physics, Institute of Physics, Budapest University of Technology and Economics, Müegyetem rakpart 3, H-1111 Budapest, Hungary

## Abstract

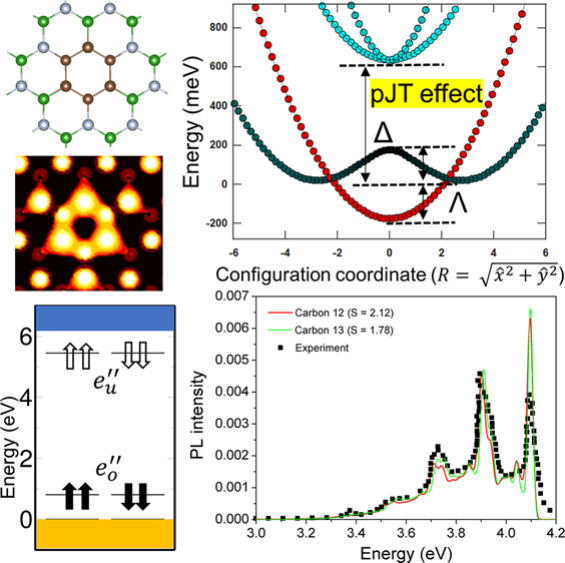

Ultraviolet (UV) quantum emitters in hexagonal boron nitride (hBN) have generated considerable interest due to their outstanding optical response. Recent experiments have identified a carbon impurity as a possible source of UV single-photon emission. Here, on the basis of first-principles calculations, we systematically evaluate the ability of substitutional carbon defects to develop the UV color centers in hBN. Of 17 defect configurations under consideration, we particularly emphasize the carbon ring defect (6C), for which the calculated zero-phonon line agrees well the experimental 4.1 eV emission signal. We also compare the optical properties of 6C with those of other relevant defects, thereby outlining the key differences in the emission mechanism. Our findings provide new insights into the strong response of this color center to external perturbations and pave the way to a robust identification of the particular carbon substitutional defects by spectroscopic methods.

Single-point defects in two-dimensional (2D) hexagonal boron nitride (hBN) play a vital role in the optical properties of the host and hold great promise for quantum information technologies and integrated quantum nanophotonics.^1−8^ In particular, color centers in hBN are responsible for ultrabright single-photon emission at room temperature with a wide range of emission wavelengths.^8,9^ Recent experiments demonstrated the versatile properties of the defect emitters in 2D hBN, such as strain- and electric field-dependent emission,^5,10−12^ high stability under high pressure and temperature,^13−15^ and initialization and readout of a spin state through optical pumping.^2,16^ Other studies have shown a successful engineering and coherent control of a single spin in hBN,^3^ while room-temperature initialization and readout have also been realized.^2,16^ Of several photoluminescence (PL) signals from the color centers in hBN, multicolor single-photon emissions have been detected around 1.6–2.2 eV. Dozens of studies have been performed to determine the possible origin based on simple defect configurations.^1−5,8,17,18^ In addition, a strong ultraviolet (UV) emission at close to ∼4.1 eV has received a great deal of attention.^19−23^ The single-photon emission associated with these bands indicates that it should originate from a point defect.^6,24^ However, despite various attempts, the atomistic origin of the UV emission in hBN is still under debate. In particular, due to the similarities with the carbon-doped hBN samples (mostly due to the PL lifetime of ∼1.1 ns^19,25^), carbon is thought to contribute to the formation of the PL signal.^21,26^ Despite the fact that some of the proposed configurations exhibit excitation energies around 4 eV,^17,21,23,27−30^ many of their key properties, including the stability, electronic configuration, and vibronic properties, were not considered. Recently, additional lines were observed in the range of 4.1–4.2 eV and isotopically controlled carbon doping is employed to determine the role of the carbon impurity in the 4.1 eV emission.^23^ In particular, the additional lines, distinct from the previous 4.1 eV emission, show strong PL intensity with a clear temperature dependency.^15^ These findings motivated us to carry out a systematic theoretical study to reveal the role of substitutional carbon defects in the formation of the UV single-photon emitters in hBN.

In this paper, we analyze 17 configurations of substitutional carbon defects and systematically address their thermodynamic properties. Among those, we identify a six-carbon ring defect, in which the carbon atoms substitute one BN honeycomb of hBN lattice, as one stable defect configuration. It is noteworthy that this defect has already been unambiguously identified via annular dark field scanning transmission electron microscopy (ADF-STEM)^31,32^ and can be intentionally introduced into the lattice with atomic precision by the focused electron beam.^32^ We show that this color center emits light due to strong electron coupling with *E*-phonon modes, caused by the product Jahn–Teller effect. More specifically, the respective symmetry lowering is found to activate a forbidden transition through an intensity borrowing mechanism from a higher-lying bright state. We further calculate the zero-phonon line (ZPL) energy, luminescence spectrum, and radiative lifetime and found them to be in excellent agreement with the experimental observations for the 4.1 eV emission. In addition, we discuss the possibilities of distinguishing between different carbon configurations on the basis of the ^13^C isotopic shift in ZPL and sideband and on the basis of a different response to the applied strain.

The calculations were performed on the basis of the spin-polarized density functional theory (DFT) within the Kohn–Sham scheme as implemented in the Vienna *ab initio* simulation package (VASP).^33,34^ A standard projector-augmented wave (PAW) formalism^35,36^ was applied to accurately describe the spin density of valence electrons close to nuclei. The screened hybrid density functional of Heyd, Scuseria, and Ernzerhof (HSE)^37^ was used to optimize the structure and calculate the electronic properties.

The calculations with the second-order approximate coupled cluster singles and doubles model (CC2)^38^ and the algebraic diagrammatic construction method [ADC(2)]^39^ were performed with the Turbomole code.^40,41^ The results of time-dependent (TD) DFT and *n*-electron valence state perturbation theory [NEVPT2(4,4)]^42^ were obtained with the ORCA code.^43^ We used the cc-pVDZ basis set^44^ and considered the PBE0 density functional^45^ for TDDFT. The periodic TD-PBE0 calculations were performed with Quantum Espresso.^46^ The details of modeling, calculation parameters, and the computation of formation energies together with the charge correction^47^ are discussed in Supplementary Note 1.

First, we systematically analyzed the thermodynamic properties of the carbon defects in hBN. Because the experimental PL signal features a short radiative lifetime, we focused on only those arrangements in which the carbon atoms are closely packed within a single honeycomb. The delocalization of defect orbitals should naturally decrease the excitation energy (see Figure 2 of the Supporting Information); therefore, larger defect complexes were not considered. The resulting structures of 17 distinct C configurations are shown in Figure 1a. For those, we evaluated the formation energy diagrams and charge transition levels (CTLs), which are plotted in Figure 1b and Figure 1 of the Supporting Information. The formation energies for the defects with an unequal amount of substituted B and N can be largely decreased by selecting the appropriate growth conditions. However, for a given number of carbon atoms, we always observed that the most stable configurations represent the confined C clusters, where the carbon atoms are arranged in a continuous chain. Importantly, to prevent a photoionization process, a UV quantum emitter should maintain a stable charge state. This condition is observed for the defects with an even number of carbon atoms (namely, C_N_C_B_, 2C_NB_, 4C_chain_, 4C_pair_, and 6C); they possess a highly stable neutral charge state over the energy range, exceeding the ionization threshold. By contrast, the defects with an odd number of carbons rapidly change their charge states across the formation energy diagrams because of their radical nature. Our calculations provide a low formation energy of 2.17 eV for carbon dimer C_N_C_B_, which is quite consistent with the previous reports.^27,48^ In addition, the formation energy of the 6C ring is found to be 1.2 eV larger than that for the dimer (0.5 eV with the PBE^49^ functional) and this is the second lowest formation energy. Considering carbon defects may be created by kinetic processes in experiments, for the binding energy discussed in Supplementary Note 1, the 6C ring has the largest binding energy^50^ among the considered carbon clusters, which means the defect will agglomerate if it can diffuse. Having identified the 6C ring defect as a stable defect configuration, we now focus on its structural and electronic properties. In the neutral charge state, the ground state configuration of the defect embedded in the hBN layer is a closed-shell singlet, and it exhibits *D*_3*h*_ symmetry. The electronic structure of hBN with the 6C ring defect is shown in Figure 2; it features two pairs of degenerate e″ orbitals where two e″ orbitals fall close to the valence band maximum, fully occupied by four electrons, and the other two fall close to the conduction band minimum. The electronic configuration reads as |e_o*x*_″e_o*y*_″e_u*x*_″e_u*y*_″⟩, where o and u indicate the occupied and unoccupied states, respectively. This leads to the ^1^*A*_1_^′^ symmetry of the ground state.

**Figure 1:**
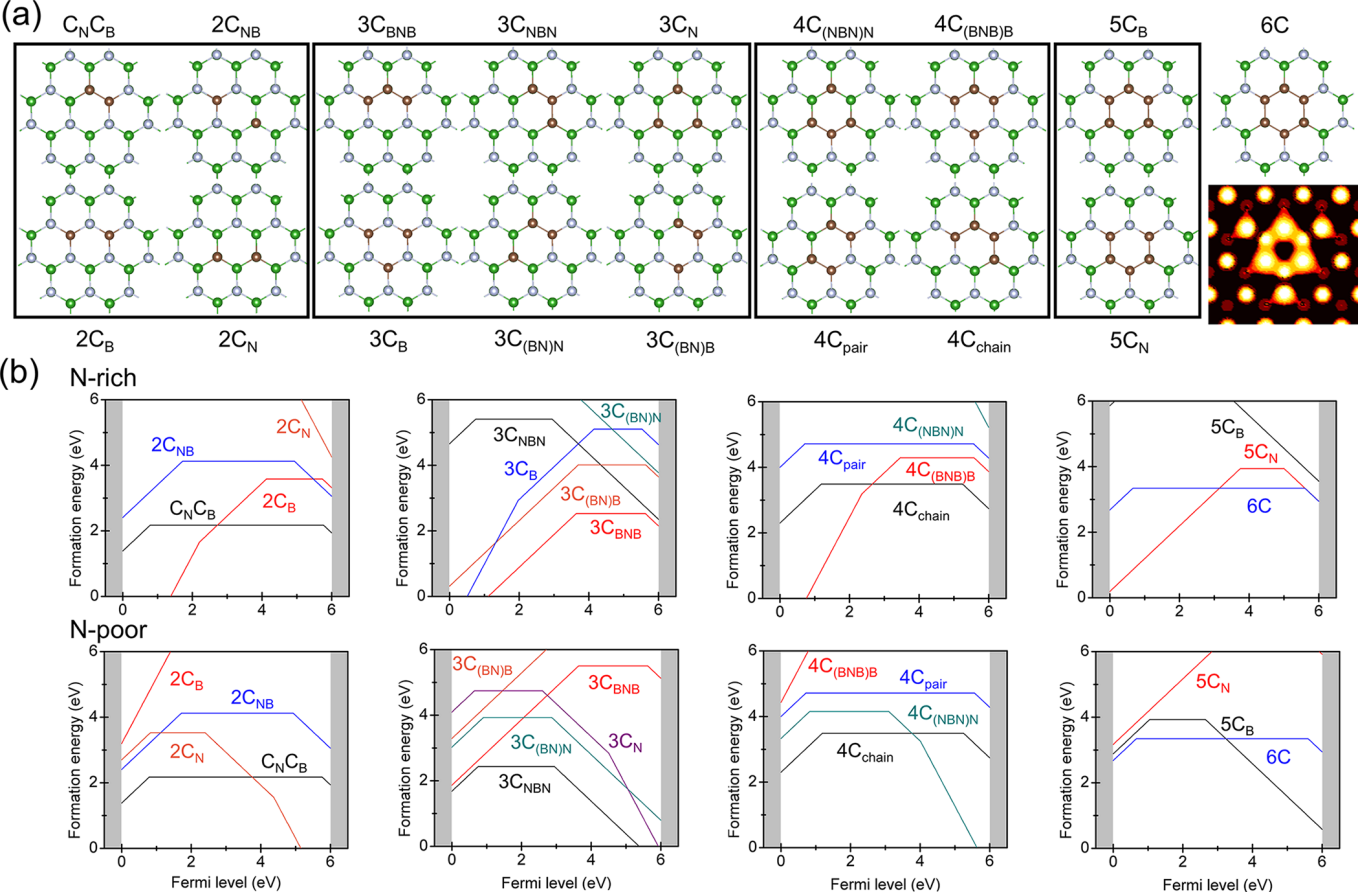
(a) Different carbon defects we considered here and a simulated scanning tunneling microscopy image for the 6C defect. (b) Calculated formation energy vs Fermi level under N-rich and N-poor conditions. The gray color depicts the band edge.

**Figure 2:**
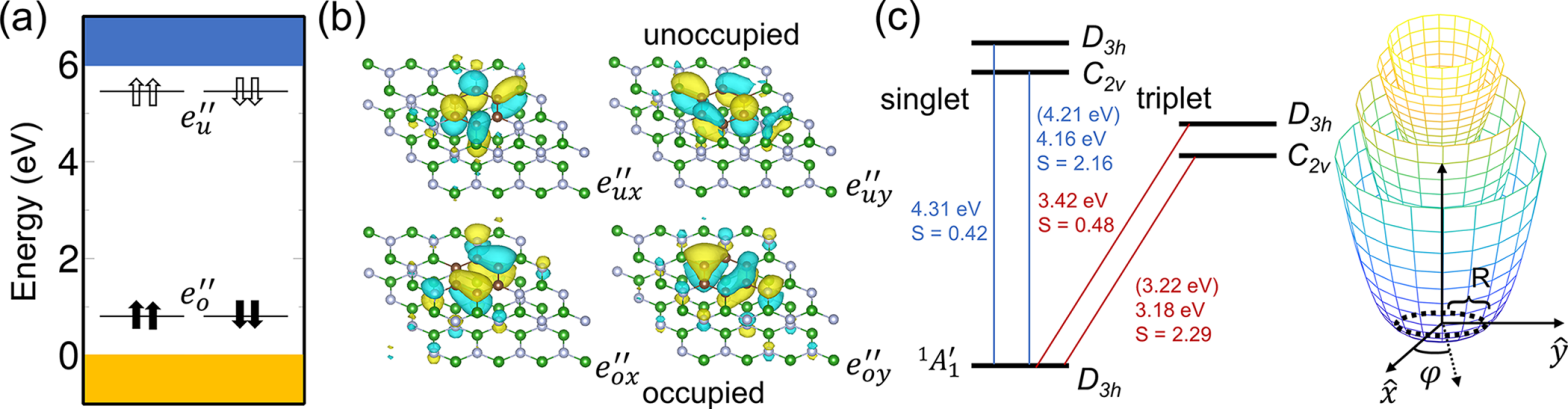
(a) Single-particle energy level of the carbon ring defect in the ground state. The subscripts *o* and *u* indicate the occupied and unoccupied defect states, respectively, while the arrows denote the spin directions. (b) Wave function isosurface of defect levels. (c) Energy diagram of the optical transition with the zero-phonon line (ZPL) and Huang–Rhys (HR) factor calculated with density functional theory. The values in parentheses are the corrected ZPL with the product Jahn–Teller (pJT) effect. The right schematic figure represents the four-layer APES of the pJT effect. The dashed line is the energetically global minimum loop.

From the group theory analysis, the electronic transitions between the e orbitals give rise to four excited states in both singlet and triplet manifolds, expressed as follows:
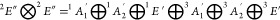
Due to the high degeneracy of the defect orbitals in *D*_3*h*_ symmetry, each excited state represents a combination of two Slater determinants (see Supplementary Note 2). Furthermore, each of the single-electron transitions leads to the Jahn–Teller instability for both occupied and empty defect orbitals; this is achieved via a coupling to a quasi-localized *E* vibration mode and is known as a product Jahn–Teller (pJT) effect.^51−53^ Thus, the total Hamiltonian, which accounts for both electronic correlation and pJT, is given as
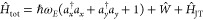
1where *a*_*x*_, *a_y_*, *a*_*x*_^†^, and *a*_*y*_^†^ are ladder operators for creating or annihilating the *E* phonon mode in the two-dimensional space while the first term is the vibrational potential energy of the system. *Ŵ* is the electronic Hamiltonian, and *Ĥ*_JT_ is the JT part.

To solve the *Ĥ*_tot_, we first construct the *Ŵ*. Here, the single determinants, which constitute the wave functions in eq 1, are shown in panels a and d of Figure 3. In *D*_3*h*_ symmetry, the four single determinants form two double-degenerate branches with *E*_d_(|e_o*x*_″e_u*x*_″⟩) = *E*_d_(|e_o*y*_″e_u*y*_″⟩) and *E*_d_(|e_o*x*_″e_u*y*_″⟩) = *E*_d_(|e_o*y*_″e_u*x*_″⟩), where *E*_d_ is the total energy of the (diabatic) state. In the singlet manifold, e_o*x*_″ → e_u*x*_″ (or e_o*y*_″ → e_u*y*_″) configurations are stabilized over 41 meV by the exchange interaction [so that *E*_d_(|e_o*x*_″e_u*y*_″⟩) is lower than *E*_d_(|e_o*x*_″e_u*x*_″⟩)], while their order is reversed for the triplets.

**Figure 3:**
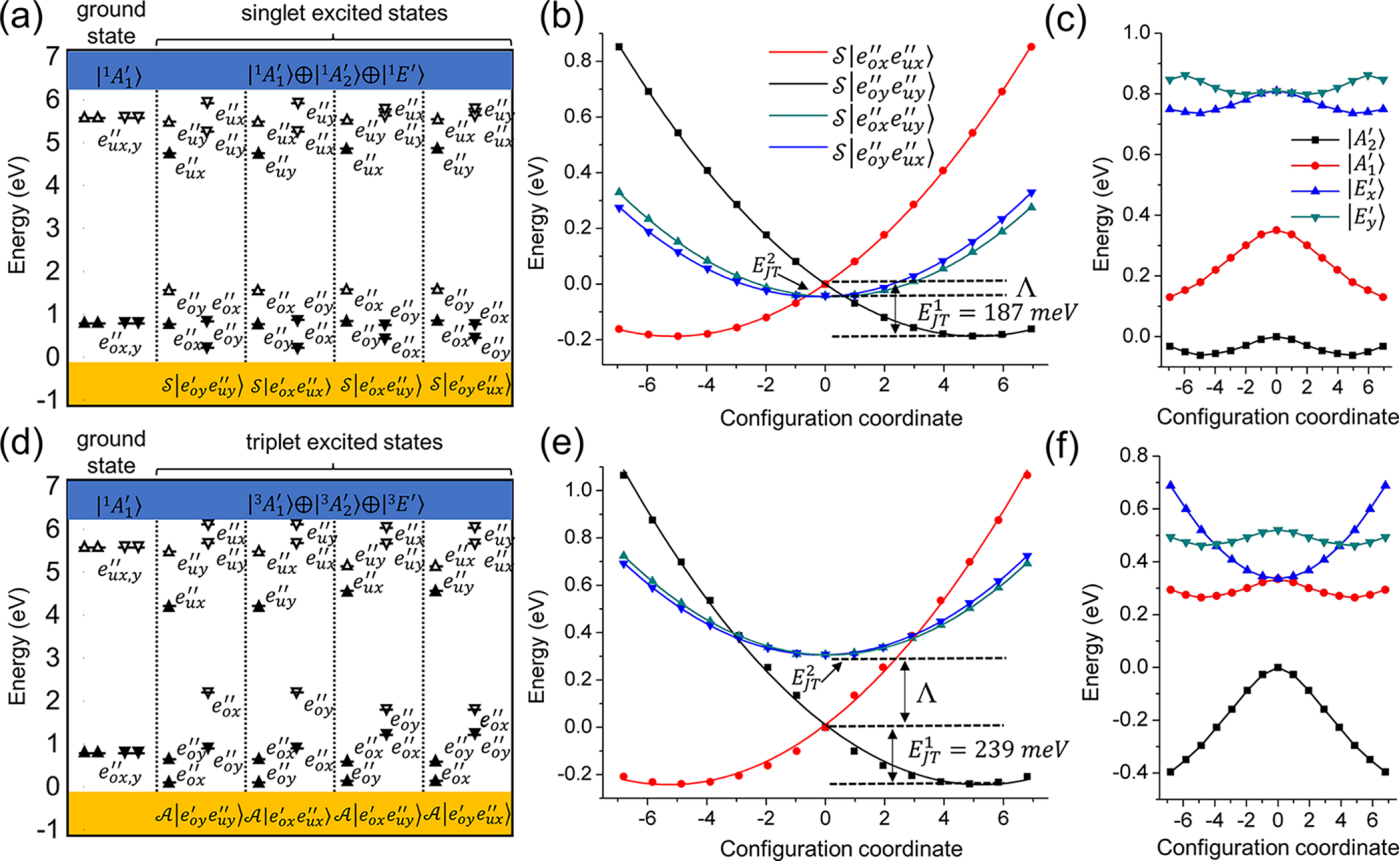
Single-particle energy level diagram of the carbon ring defect for (a) singlet and (d) triplet excited states. The filled and empty arrows indicate the occupied and empty states with up and down spin directions, respectively. (b and e) Calculated APES for the singlet and triplet states, respectively. The dots are from DFT results, and the solid line is fitted on the basis of the pJT model. The standard deviation is <3%. X = 0 is the geometry with *D*_3*h*_ symmetry, and the energy minima could be achieved by removing the symmetry restriction. (c and f) Energy diagrams for the four states with the TDDFT method for the singlet and triplet states, respectively. The coordinates are built on DFT optimization. The pJT effect is not included here.

Due to the complex nature of the excited states, the electronic Hamiltonian needs to be defined by using a robust method for the excited states. To this end, we compute the excitation energies of the 6C defect by CC2, focusing on a representative flake model. These calculations were assisted by TDDFT to access the transition properties, as well as by two other post-Hartree fock methods (SOS-ADC2 and NEVPT2). The resulting (vertical) excitation energies, obtained at the HSE geometry, are summarized in Table 3 of the Supporting Information. Here, we found that all of the approaches consistently predict the appearance of the localized excited states in the energy range between 4 and 5 eV. It is noteworthy that, at the high symmetry point, the two lowest *A*_1_^′^ and *A*_2_^′^ states are dark, while the transitions to *E*′ are optically allowed, whic is evident because of the value of the oscillator strength (∼0.93 atomic unit). From these calculations, using the definition from refs (51) and (52), the electronic Hamiltonian is expressed as follows (see Supplementary Note 3)
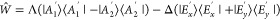
2where *A*_1_^′^ and *A*_2_^′^ are nondegenerate states and *E*′ is a double degenerate state. Here, Λ and Δ indicate the static electronic correlation energies for the *A*_1,2_^′^ and *E*′ states, respectively. The values obtained by TDDFT are −175.5 and −634.5 meV, respectively, for the singlets and 260.5 and 74.5 meV, respectively, for the triplets.

Having defined *Ŵ*, we now focus on the pJT Hamiltonian, given as
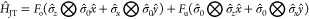
3where σ̂_*z*_ = |e_*x*_⟩⟨e_*x*_| – |e_*y*_⟩⟨e_*y*_| and σ̂_*x*_ = |e_*x*_⟩⟨e_*y*_| + |e_*y*_⟩⟨e_*x*_| are Pauli matrices; σ̂_0_ is the unit matrix, and σ̂_0_ = |e_*x*_⟩⟨e_*x*_| + |e_*y*_⟩⟨e_*y*_|. *F*_o_ and *F*_u_ are the electron–phonon coupling coefficients, and the major effect of the strong electron–phonon coupling is to drive the excited states out of *D*_3*h*_ symmetry to a lower *C*_2*v*_ symmetry by elongating two of six C–C bonds. The JT energies, denoted as *E*_JT_^1^ and *E*_JT_^2^ for |e_o*x*_″e_u*x*_″⟩ and |e_o*x*_″e_u*y*_″⟩, respectively, are determined by fitting the adiabatic potential energy surfaces (APES) from *ab initio* results, as shown in Figure 3. We found that the JT effect is much more significant for |e_o*x*_″e_u*x*_″⟩ than for |e_o*x*_″e_u*y*_″⟩, which yields the negligible *E*_JT_^2^. More specifically, the values of *E*_JT_^1^ are 187 and 239 meV for the singlets and triplets, respectively, while the *E*_JT_^2^ values are only 0.46 and 0.14 meV, respectively. The effective vibration energy *ℏω*_E_ is then deduced from the lowest branch of the APES parabola in dimensionless generalized coordinates. The detailed construction of the pJT Hamiltonian is provided in Supplementary Note 4.

The solutions for the total Hamiltonian from eq 1 that incorporate the vibrational and electronic parts for the singlet and triplet states are plotted in Figure 4. For the singlets in *D*_3*h*_ symmetry, the states appear in the following order: E(*A*_2_^′^) < E(*A*_1_^′^) < E(*E*′). *A*_2_^′^ shows no sign of the JT instability or a mixture with *E*′; thus, it maintains a high-symmetry configuration and remains dark along the configuration coordinate. By contrast, when the system is driven out of *D*_3*h*_ symmetry, the mixing between *A*_1_^′^ and *E*′ is apparent.

**Figure 4:**
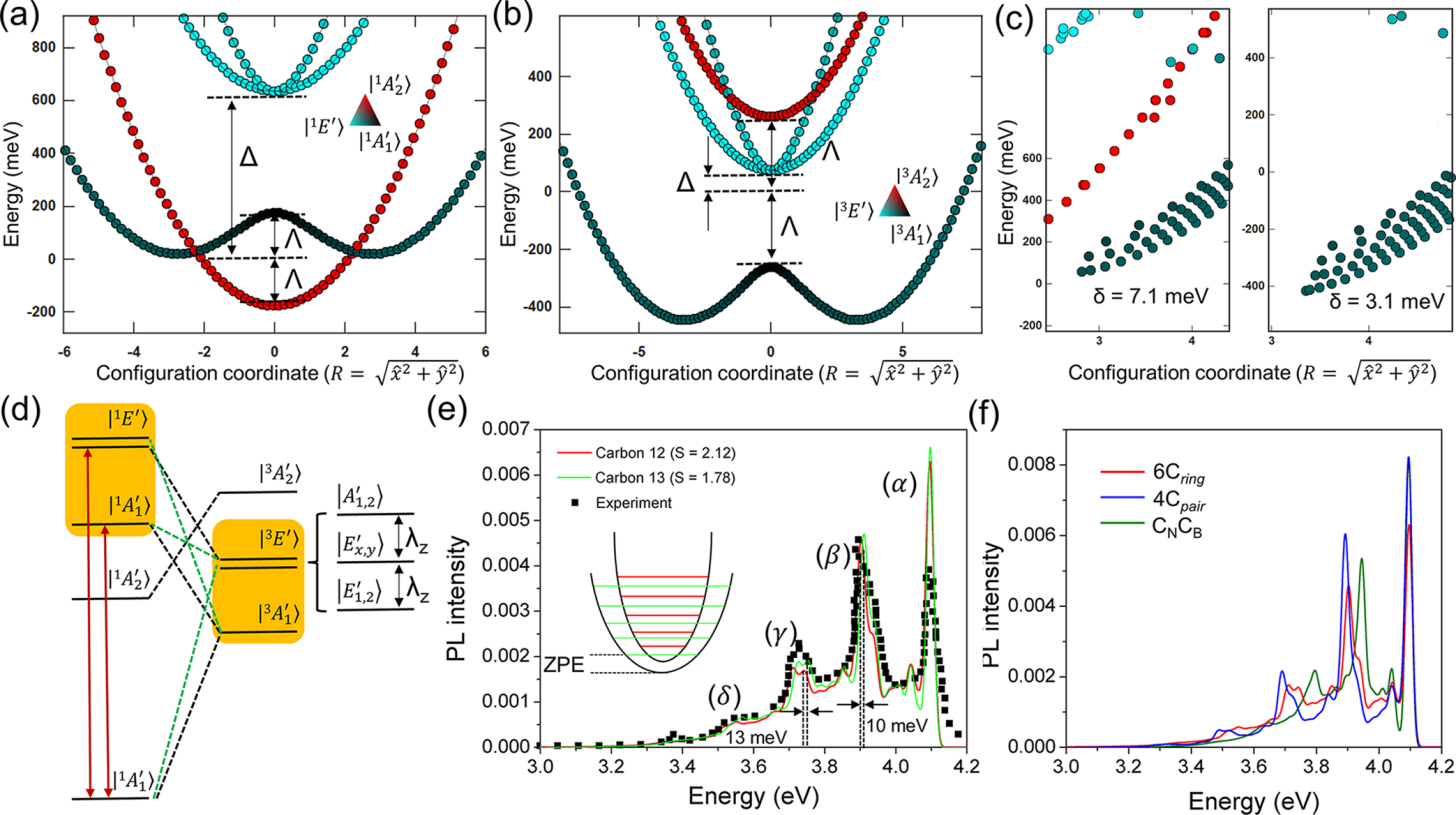
Eigenvalues for the total Hamiltonian of the system in one dimension (*Y* = 0) for (a) singlet and (b) triplet states. Data for pure states *A*_1_^′^, *A*_2_^′^, and *E*′ are depicted with black, red, and cyan dots, respectively. The lowest APES branch is a mixed state of *A*_1_^′^ and *E*′. (c) Polaronic eigenstates for the (left) singlet and (right) triplet with full rotation. The second-order pJT strength could be estimated by the energy splitting between the two lowest eigenvalues. (d) Schematic energy diagram of the electronic states and possible ISC transitions. The black dashed line links states with the same representation in different spin manifolds. The green line links states enabled by pJT-induced mixing that happens between states labeled in orange. (e) Simulated PL spectrum (red) and experimental data (black dots). The PSB of isotope ^13^C is also shown. The ZPL position is aligned by 0.08 eV to match the first peak in the PSB. The Gaussian broadening is 10 meV. Four peaks can be identified at 4.095, 3.905, 3.711, and 3.551 eV, which are consistent with experimental observation. The inset is the schematic coordinate diagram of the isotopic effect. (f) Simulated PL spectrum of the dimer (C_N_C_B_), 4C_pair_, and 6C ring where the ZPL energies are aligned for the sake of comparison of PSBs.

A direct diagonalization of the total Hamiltonian with the pJT and electronic part ⟨Φ̃|*Ĥ*_tot_|Φ̃⟩ is shown in Figure 4c (see Supplementary Note 5). A converged solution demonstrates that the lowest eigenstate contains 68% of the *Ã*_1_^′^ component in the singlet manifold (and 63% in the triplet manifold). The energy splittings between the lowest two eigenvalues are 7.1 and 3.1 meV for the singlets and triplets, respectively. On the basis of the degeneracy of polaronic levels, we assigned the lowest state to *Ã*_1_^′^ and the second one to *Ẽ*′. Given that only *Ẽ*′ is bright, the process requires a thermal activation and results in strongly temperature-dependent PL emission. Furthermore, the position of the ZPL based on the full Hamiltonian is calculated as follows
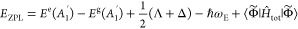
4where *E*^e^ and *E*^g^ are the energies of excited state and ground state, respectively. The computed value is 4.21 eV, which closely agrees with the experimental data.

To further support the validity of our model calculations, we approach the *A*_1_^′^ geometry by TDDFT and CC2. The robust CC2 approach predicts a decrease in the symmetry to *C*_2*v*_, while the TDDFT method preserves the *D*_3*h*_ symmetry. Here, the energy gap between *A*_1_^′^ and *E*′ reflects the magnitude of the electronic coupling between the respective diabatic states. In the case of TDDFT, the value (223 meV) is considerably larger than that from CC2 (178 meV); this points to a strong coupling regime, where two diabats develop a single minima on the APES.^54^ For the 6C defect, this relaxation is particularly important, because the coupling to the *E* phonon mode enables the intensity borrowing from the allowed *E*′; otherwise, the *A*_1_^′^ state remains optically forbidden.

To clarify the discrepancy between TDDFT and CC2 for the excited state geometry, we performed the TD-PBE0 calculations in a periodic monolayer. The results, shown in Table 4 of the Supporting Information, indicate that the flake model provides a reasonable description of the vertical spectrum relative to the periodic structure. However, while the difference between the *A*_2_^′^ energies is only 37 meV, it gradually increases to 298 meV for *E*′. Indeed, in this case, the electronic coupling between *A*_1_^′^ and *E*′ decreases to a much smaller value of 135 meV, and therefore, the stabilization of the *C*_2*v*_ configuration is expected. We associate this behavior with the quantum confinement effect, which appears to be more harmful for TDDFT than for the wave function-based methods.

The simulated PL spectrum including the pJT distortion is shown in Figure 4e. Here, four prominent peaks in the phonon sideband with an averaged energy space of 180.3 meV perfectly match the experimental PL spectrum.^19^ From these calculations, we also determine a Huang–Rhys (HR) factor, *S*, of 2.16, which closely agrees with the experimental results (*S* = 1–2). In addition, with the CC2 approach, we obtained a HR factor of 1.3 for the heteroatoms forming the flake. It is noteworthy that, at the relaxed *A*_1_^′^ geometry, the CC2 approach predicts that the wave function is governed by a single determinant with a relative contribution of 83%. This justifies the application of the ΔSCF for computing the vibronic sideband of *A*_1_^′^.

Next, we evaluate the radiative lifetimes on the basis of the following expression
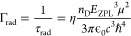
5where ϵ_0_ is the vacuum permittivity, *ℏ* is the reduced Planck constant, *c* is the speed of light, *n*_D_ = 2.5 is the refractive index of hBN at ZPL energy *E*_ZPL_, μ is the optical transition dipole moment, and η is the fraction of *E*′ in the polaronic state. The symmetry lowering makes *Ẽ*′ less bright (see Table 5 of the Supporting Information), yielding a τ_rad_ of 1.54 ns at room temperature (2 ns at 150 K for the SPE experiment^6^). This value is temperature-dependent considering the thermal occupation of *Ẽ*′. Nonetheless, it is very close to the observed value of ∼1.1 ns.^19^

A nonradiative transition occurs between *A*_1_^′^ and the lower-lying *A*_2_^′^ in the singlet manifold. This process could bleach the fluorescence if it is faster than the emission. In a low-temperature limit, the computed rate is 509 MHz (1.98 ns), which is slower than the radiative rate mentioned above. The optimal quantum efficiency for the defect is 52% at 300 K. However, this is influenced by the temperature, which can change the distribution between the dark and bright polaronic states, as shown in Supplementary Notes 6 and 7. We note that the nonradiative decay via phonons from the singlet *A*_2_^′^ toward the ground state is very slow due to the large gap between the two; thus, recombination of hot charge carriers via a two-photon absorption process is the likely process for obtaing the ground state once the electron is scattered to the dark singlet *A*_2_^′^ state.

Finally, after identifying the 6C defect as a promising candidate for UV emission, we compare its properties with those of 4C and C_N_C_B_. While the C_N_C_B_ defect was described elsewhere,^27^ for 4C_pair_ we computed a ZPL of ∼4.4 eV and a HR factor of 1.9. As demonstrated in Figure 4f, all three defects exhibit a remarkably similar phonon sideband. The minor differences between those are seen in the intensities of the replicas at the lower energies. These findings are in line with a recent experimental work in which a continuous distribution of ZPL lines around 4.1 eV^23^ is observed. The similarities of PL features among these three defects indicate that other experimental techniques are needed to distinguish among those. In particular, in Supplementary Note 8, we show that the three defects demonstrate a slightly different blue shift with respect to the content of isotope ^13^C. Moreover, as discussed in Supplementary Note 9, we found a striking difference for these defects when considering a response to the applied strain. More specifically, the optical intensity for 6C is found to be largely affected by the uniaxial strain, but in the case of C_N_C_B_, only a weak effect is observed. Therefore, although different carbon pairs are experimentally feasible, it is essentially the 6C defect that permits a high sensitivity of the signal to the external perturbations.

In summary, on the basis of an extensive theoretical investigation, we explored the potential of substitution of carbon defects for developing UV single-photon sources in hBN. We found that carbon atoms are preferentially arranged into chains, which are stabilized to the formation of energetically favorable C–C bonds. Of those defect configurations, we identified several potential candidates for UV emission, including C_N_C_B_, 4C, and 6C defects, because they feature a photostable (neutral) charge state. The 6C defect of which configuration was observed in the experiments exhibits a highly nontrivial emission mechanism in which the second excited state is optically activated by the product Jahn–Teller effect. More specifically, the ZPL is computed at 4.21 eV and the HR factor is found to be 2.1. The simulated PL spectrum shows the phonon replicas with an energy spacing of 180 meV. The upper limit of the estimated radiative lifetime is ∼1.17 ns. All of these properties closely resemble the PL signal that is present in many hBN samples. Given the relatively low formation energy and complete agreement with the experimental measurements, these results outline the 6C defect as a plausible source of the observed UV emission. We infer that the 4.1 eV PL signal likely appears as a commutative effect from different types of point defects. Furthermore, it is likely that the 6C ring defect is responsible for the temperature^15^ and strain dependency of the emission from the family of 4.1 eV emitters.

## Supporting Information Available

Calculation details, the two-hole wave functions with *D*_3*h*_ symmetry, electronic term *Ŵ* Hamiltonian, derivation of the product Jahn–Teller Hamiltonian, four-layer product Jahn–Teller system and the lowest polaronic state, intersystem crossing and nonradiative decay from |*A*_1_^′^⟩ to |*A*_2_^′^⟩; temperature-dependent quantum yield, phonon properties related to carbon defects, and effect of strain on C_N_C_B_ and 6C (PDF)
Additional experimental data (ZIP)

## References

[ref1] Tran T. T.; Bray K.; Ford M. J.; Toth M.; Aharonovich I. Quantum emission from hexagonal boron nitride monolayers. Nat. Nanotechnol. 2016, 11, 37–41. 10.1038/nnano.2015.242. 26501751

[ref2] Gottscholl A.; Kianinia M.; Soltamov V.; Orlinskii S.; Mamin G.; Bradac C.; Kasper C.; Krambrock K.; Sperlich A.; Toth M.; et al. Initialization and read-out of intrinsic spin defects in a van der waals crystal at room temperature. Nat. Mater. 2020, 19, 540–545. 10.1038/s41563-020-0619-6. 32094496

[ref3] Chejanovsky N.; Mukherjee A.; Geng J.; Chen Y.-C.; Kim Y.; Denisenko A.; Finkler A.; Taniguchi T.; Watanabe K.; Dasari D. B. R.; et al. Single-spin resonance in a van der waals embedded paramagnetic defect. Nat. Mater. 2021, 20, 1079–1084. 10.1038/s41563-021-00979-4. 33958771

[ref4] Mendelson N.; Chugh D.; Reimers J. R.; Cheng T. S.; Gottscholl A.; Long H.; Mellor C. J.; Zettl A.; Dyakonov V.; Beton P. H.; et al. Identifying carbon as the source of visible single-photon emission from hexagonal boron nitride. Nat. Mater. 2021, 20, 321–328. 10.1038/s41563-020-00850-y. 33139892

[ref5] Hayee F.; Yu L.; Zhang J. L.; Ciccarino C. J.; Nguyen M.; Marshall A. F.; Aharonovich I.; Vučković J.; Narang P.; Heinz T. F.; et al. Revealing multiple classes of stable quantum emitters in hexagonal boron nitride with correlated optical and electron microscopy. Nat. Mater. 2020, 19, 534–539. 10.1038/s41563-020-0616-9. 32094492

[ref6] Bourrellier R.; Meuret S.; Tararan A.; Stéphan O.; Kociak M.; Tizei L. H.; Zobelli A. Bright UV single photon emission at point defects in h-bn. Nano Lett. 2016, 16, 4317–4321. 10.1021/acs.nanolett.6b01368. 27299915

[ref7] Bommer A.; Becher C. New insights into nonclassical light emission from defects in multi-layer hexagonal boron nitride. Nanophotonics 2019, 8, 2041–2048. 10.1515/nanoph-2019-0123.

[ref8] Tran T. T.; Elbadawi C.; Totonjian D.; Lobo C. J.; Grosso G.; Moon H.; Englund D. R.; Ford M. J.; Aharonovich I.; Toth M. Robust multicolor single photon emission from point defects in hexagonal boron nitride. ACS Nano 2016, 10, 7331–7338. 10.1021/acsnano.6b03602. 27399936

[ref9] Sajid A.; Ford M. J.; Reimers J. R. Single-photon emitters in hexagonal boron nitride: a review of progress. Rep. Prog. Phys. 2020, 83, 044501 10.1088/1361-6633/ab6310. 31846956

[ref10] Grosso G.; Moon H.; Lienhard B.; Ali S.; Efetov D. K.; Furchi M. M.; Jarillo-Herrero P.; Ford M. J.; Aharonovich I.; Englund D. Tunable and high-purity room temperature single-photon emission from atomic defects in hexagonal boron nitride. Nat. Commun. 2017, 8, 705 10.1038/s41467-017-00810-2. 28951591 PMC5615041

[ref11] Mendelson N.; Doherty M.; Toth M.; Aharonovich I.; Tran T. T. Strain-induced modification of the optical characteristics of quantum emitters in hexagonal boron nitride. Adv. Mater. 2020, 32, 1908316 10.1002/adma.201908316. 32270896

[ref12] Noh G.; Choi D.; Kim J.-H.; Im D.-G.; Kim Y.-H.; Seo H.; Lee J. Stark tuning of single-photon emitters in hexagonal boron nitride. Nano Lett. 2018, 18, 4710–4715. 10.1021/acs.nanolett.8b01030. 29932664

[ref13] Xue Y.; Wang H.; Tan Q.; Zhang J.; Yu T.; Ding K.; Jiang D.; Dou X.; Shi J.-j.; Sun B.-q. Anomalous pressure characteristics of defects in hexagonal boron nitride flakes. ACS Nano 2018, 12, 7127–7133. 10.1021/acsnano.8b02970. 29957923

[ref14] Kianinia M.; Tawfik S. A.; Regan B.; Tran T. T.; Ford M. J.; Aharonovich I.; Toth M. CLEO: Applications and Technology; 2017; p JTu5A-24.

[ref15] Vokhmintsev A.; Weinstein I. Temperature effects in 3.9 ev photoluminescence of hexagonal boron nitride under band-to-band and subband excitation within 7–1100 k range. J. Lumin. 2021, 230, 117623 10.1016/j.jlumin.2020.117623.

[ref16] Gottscholl A.; Diez M.; Soltamov V.; Kasper C.; Sperlich A.; Kianinia M.; Bradac C.; Aharonovich I.; Dyakonov V. Room temperature coherent control of spin defects in hexagonal boron nitride. Sci. Adv. 2021, 7, eabf3630 10.1126/sciadv.abf3630. 33811078 PMC11059373

[ref17] Weston L.; Wickramaratne D.; Mackoit M.; Alkauskas A.; Van de Walle C. Native point defects and impurities in hexagonal boron nitride. Phys. Rev. B 2018, 97, 214104 10.1103/PhysRevB.97.214104.

[ref18] Ivády V.; Barcza G.; Thiering G.; Li S.; Hamdi H.; Chou J.-P.; Legeza Ö.; Gali A. Ab initio theory of the negatively charged boron vacancy qubit in hexagonal boron nitride. npj Comput. Mater. 2020, 6, 41 10.1038/s41524-020-0305-x.

[ref19] Museur L.; Feldbach E.; Kanaev A. Defect-related photoluminescence of hexagonal boron nitride. Phys. Rev. B 2008, 78, 155204 10.1103/PhysRevB.78.155204.

[ref20] Watanabe K.; Taniguchi T.; Kanda H. Direct-bandgap properties and evidence for ultraviolet lasing of hexagonal boron nitride single crystal. Nat. Mater. 2004, 3, 404–409. 10.1038/nmat1134. 15156198

[ref21] Du X.; Li J.; Lin J.; Jiang H. The origin of deep-level impurity transitions in hexagonal boron nitride. Appl. Phys. Lett. 2015, 106, 021110 10.1063/1.4905908.

[ref22] Vuong T.; Cassabois G.; Valvin P.; Ouerghi A.; Chassagneux Y.; Voisin C.; Gil B. Phonon-photon mapping in a color center in hexagonal boron nitride. Phys. Rev. Lett. 2016, 117, 097402 10.1103/PhysRevLett.117.097402. 27610882

[ref23] Pelini T.; Elias C.; Page R.; Xue L.; Liu S.; Li J.; Edgar J.; Dréau A.; Jacques V.; Valvin P.; et al. Shallow and deep levels in carbon-doped hexagonal boron nitride crystals. Phys. Rev. Mater. 2019, 3, 094001 10.1103/PhysRevMaterials.3.094001.

[ref24] Tan Q.-H.; Xu K.-X.; Liu X.-L.; Guo D.; Xue Y.-Z.; Ren S.-L.; Gao Y.-F.; Dou X.-M.; Sun B.-Q.; Deng H.-X. Ultraviolet to near-infrared single photon emitters in hbn. arXiv 2019, 1908.06578.

[ref25] Era K.; Minami F.; Kuzuba T. Fast luminescence from carbon-related defects of hexagonal boron nitride. J. Lumin. 1981, 24, 71–74. 10.1016/0022-2313(81)90223-4.

[ref26] Uddin M.; Li J.; Lin J.; Jiang H. Probing carbon impurities in hexagonal boron nitride epilayers. Appl. Phys. Lett. 2017, 110, 182107 10.1063/1.4982647.

[ref27] Mackoit-Sinkevičienė M.; Maciaszek M.; Van de Walle C. G.; Alkauskas A. Carbon dimer defect as a source of the 4.1 ev luminescence in hexagonal boron nitride. Appl. Phys. Lett. 2019, 115, 212101 10.1063/1.5124153.

[ref28] Korona T.; Chojecki M. Exploring point defects in hexagonal boron-nitrogen monolayers. Int. J. Quantum Chem. 2019, 119, e25925 10.1002/qua.25925.

[ref29] Jara C.; Rauch T.; Botti S.; Marques M. A.; Norambuena A.; Coto R.; Castellanos-Águila J.; Maze J. R.; Munoz F. First-principles identification of single photon emitters based on carbon clusters in hexagonal boron nitride. J. Phys. Chem. A 2021, 125, 1325–1335. 10.1021/acs.jpca.0c07339. 33554602

[ref30] Hamdi H.; Thiering G.; Bodrog Z.; Ivády V.; Gali A. Stone-wales defects in hexagonal boron nitride as ultraviolet emitters. npj Comput. Mater. 2020, 6, 178 10.1038/s41524-020-00451-y.

[ref31] Krivanek O. L.; Chisholm M. F.; Nicolosi V.; Pennycook T. J.; Corbin G. J.; Dellby N.; Murfitt M. F.; Own C. S.; Szilagyi Z. S.; Oxley M. P.; et al. Atom-by-atom structural and chemical analysis by annular dark-field electron microscopy. Nature 2010, 464, 571–574. 10.1038/nature08879. 20336141

[ref32] Park H.; Wen Y.; Li S. X.; Choi W.; Lee G.-D.; Strano M.; Warner J. H. Atomically precise control of carbon insertion into hbn monolayer point vacancies using a focused electron beam guide. Small 2021, 17, 2100693 10.1002/smll.202100693. 33960117

[ref33] Kresse G.; Furthmüller J. Efficiency of ab-initio total energy calculations for metals and semiconductors using a plane-wave basis set. Comput. Mater. Sci. 1996, 6, 15–50. 10.1016/0927-0256(96)00008-0. 9984901

[ref34] Kresse G.; Furthmüller J. Efficient iterative schemes for ab initio total-energy calculations using a plane-wave basis set. Phys. Rev. B 1996, 54, 11169 10.1103/PhysRevB.54.11169. 9984901

[ref35] Blöchl P. E. Projector augmented-wave method. Phys. Rev. B 1994, 50, 17953 10.1103/PhysRevB.50.17953. 9976227

[ref36] Kresse G.; Joubert D. From ultrasoft pseudopotentials to the projector augmented-wave method. Phys. Rev. B 1999, 59, 1758 10.1103/PhysRevB.59.1758.

[ref37] Heyd J.; Scuseria G. E.; Ernzerhof M. Hybrid functionals based on a screened coulomb potential. J. Chem. Phys. 2003, 118, 8207–8215. 10.1063/1.1564060.

[ref38] Christiansen O.; Koch H.; Jørgensen P. The second-order approximate coupled cluster singles and doubles model cc2. Chem. Phys. Lett. 1995, 243, 409–418. 10.1016/0009-2614(95)00841-Q.

[ref39] Schirmer J. Beyond the random-phase approximation: a new approximation scheme for the polarization propagator. Phys. Rev. A 1982, 26, 2395 10.1103/PhysRevA.26.2395.

[ref40] Ahlrichs R.; Bär M.; Häser M.; Horn H.; Kölmel C. Electronic structure calculations on workstation computers: the program system turbomole. Chem. Phys. Lett. 1989, 162, 165–169. 10.1016/0009-2614(89)85118-8.

[ref41] TURBOMOLE, ver. 6.4; 2017, a development of University of Karlsruhe and Forschungszentrum Harlsruhe Gmbh, 1989–2007, turbomole gmbh, since 2007.

[ref42] Angeli C.; Cimiraglia R.; Evangelisti S.; Leininger T.; Malrieu J.-P. Introduction of n-electron valence states for multireference perturbation theory. J. Chem. Phys. 2001, 114, 10252–10264. 10.1063/1.1361246.

[ref43] Neese F. Software update: the orca program system, version 4.0. Wiley Interdiscip. Rev.: Comput. Mol. Sci. 2018, 8, e1327 10.1002/wcms.1327.

[ref44] Dunning T. H. Jr. Gaussian basis sets for use in correlated molecular calculations. i. the atoms boron through neon and hydrogen. J. Chem. Phys. 1989, 90, 1007–1023. 10.1063/1.456153.

[ref45] Perdew J. P.; Ernzerhof M.; Burke K. Rationale for mixing exact exchange with density functional approximations. J. Chem. Phys. 1996, 105, 9982–9985. 10.1063/1.472933.

[ref46] Giannozzi P.; Baroni S.; Bonini N.; Calandra M.; Car R.; Cavazzoni C.; Ceresoli D.; Chiarotti G. L.; Cococcioni M.; Dabo I.; et al. Quantum espresso: a modular and open-source software project for quantum simulations of materials. J. Phys.: Condens. Matter 2009, 21, 395502 10.1088/0953-8984/21/39/395502. 21832390

[ref47] Freysoldt C.; Neugebauer J. First-principles calculations for charged defects at surfaces, interfaces, and two-dimensional materials in the presence of electric fields. Phys. Rev. B 2018, 97, 205425 10.1103/PhysRevB.97.205425.

[ref48] Maciaszek M.; Razinkovas L.; Alkauskas A. Thermodynamics of carbon point defects in hexagonal boron nitride. Phys. Rev. Mater. 2022, 6, 014005 10.1103/PhysRevMaterials.6.014005.

[ref49] Perdew J. P.; Burke K.; Ernzerhof M. Generalized gradient approximation made simple. Phys. Rev. Lett. 1996, 77, 3865 10.1103/PhysRevLett.77.3865. 10062328

[ref50] Huang P.; Grzeszczyk M.; Vaklinova K.; Watanabe K.; Taniguchi T.; Novoselov K.; Koperski M. Carbon and vacancy centers in hexagonal boron nitride. arXiv 2021, 2112.14906.

[ref51] Thiering G.; Gali A. The (*e*_*g*_ ⊗ *e*_*u*_) ⊗ *E*_*g*_ product jahn-teller effect in the neutral group-iv vacancy quantum bits in diamond. npj Comput. Mater. 2019, 5, 18 10.1038/s41524-019-0158-3.

[ref52] Ciccarino C. J.; Flick J.; Harris I. B.; Trusheim M. E.; Englund D. R.; Narang P. Strong spin-orbit quenching via the product jahn-teller effect in neutral group iv qubits in diamond. npj Quantum Mater. 2020, 5, 75 10.1038/s41535-020-00281-7.

[ref53] Qiu Q.-c. Studies of the doubly degenerate product jahn-teller system. Front. Phys. China 2007, 2, 51–54. 10.1007/s11467-007-0019-2.

[ref54] Sampaio R. N.; Piechota E. J.; Troian-Gautier L.; Maurer A. B.; Hu K.; Schauer P. A.; Blair A. D.; Berlinguette C. P.; Meyer G. J. Kinetics teach that electronic coupling lowers the free-energy change that accompanies electron transfer. Proc. Natl. Acad. Sci. U.S.A. 2018, 115, 7248–7253. 10.1073/pnas.1722401115. 29941573 PMC6048547

