# Supplementary material for: Ultraviolet Quantum Emitters in Hexagonal Boron Nitride from Carbon Clusters
Source: J Phys Chem Lett. 2022 Apr 1;13(14):3150–7. doi: 10.1021/acs.jpclett.2c00665 (PMC9014460; doi:10.1021/acs.jpclett.2c00665)
Supplement: Supplementary file 1 — jz2c00665_si_001.pdf [file jz2c00665_si_001.pdf]

Supporting Information for Ultraviolet Quantum Emitters in Hexagonal Boron Nitride from Carbon Clusters

Song Li,† Anton Pershin,† Gergő Thiering,† Péter Udvarhelyi,† and Adam Gali*,†,‡

†*Wigner Research Centre for Physics, P.O. Box 49, H-1525 Budapest, Hungary*

‡*Department of Atomic Physics, Institute of Physics, Budapest University of Technology and Economics, Műegyetem rakpart 3., H-1111 Budapest, Hungary*

E-mail: gali.adam@wigner.hu

SUPPLEMENTARY NOTE 1: Calculation details

The calculations were performed based on the spin-polarized DFT within the Kohn-Sham scheme as implemented in Vienna *ab initio* simulation package (VASP).[1,2] A standard projector augmented wave (PAW) formalism[3,4] is applied to accurately describe the spin density of valence electrons close to nuclei. The carbon defects were embedded in a 7×7 bulk supercell with 196 atoms. The atoms were fully relaxed with a plane wave cutoff energy of 450 eV until the forces acting on ions were less than 0.01 eV/Å. The Brillouin-zone was sampled by the single Γ-point scheme. The screened hybrid density functional of Heyd, Scuseria, and Ernzerhof (HSE)[5] was used to optimize the structure and calculate the electronic properties. By changing the α parameter, we modified a part of nonlocal Hartree-Fock exchange to the generalized gradient approximation of Perdew, Burke, and Ernzerhof (PBE)[6] with fraction α to adjust the calculated band gap. Here, $\alpha = 0.32$ was used which could reproduce the experimental band gap about 6 eV. The optimized interlayer distance was 3.37 Å with DFT-D3 method of Grimme.[7] The excited states were calculated by ΔSCF method.[8] The defect formation energies E_f was calculated according to the following equation,

$$\begin{aligned} E_f^q =& E_d^q - E_\mathrm{p} - n_\mathrm{C}\mu_\mathrm{C} + n_\mathrm{B}\mu_\mathrm{B} + n_\mathrm{N}\mu_\mathrm{N} + \\ & q\left(\epsilon_\mathrm{VBM}^\mathrm{p} + \epsilon_\mathrm{Fermi}\right) + E_\mathrm{corr}\left(q\right), \end{aligned} \tag{1}$$

where E_d^q is the total energy of hBN model with defect at q charge state and E_p is the total energy of hBN layer without defect. μ_C is the chemical potential of carbon and can be derived from pure graphite. For N-rich condition, the chemical potential $\mu_\mathrm{N} = 1/2E(\mathrm{N}_2)$, which is half of nitrogen gas molecule. For N-poor condition, the chemical potential μ_B is derived from pure bulk boron and $\mu_\mathrm{BN} = \mu_\mathrm{B} + \mu_\mathrm{N}$. The Fermi level ϵ_Fermi represents the chemical potential of electron reservoir and it is aligned to the valence band maximum (VBM) energy of perfect hBN, $\epsilon_\mathrm{VBM}^\mathrm{p}$. The $E_\mathrm{corr}\left(q\right)$ is the correction term for the charged system due to the existence of electrostatic interactions with periodic condition. The charge correction terms

were computed by SXDEFECTALIGN code from Freysoldt method.[9] The charge transition levels for a given defect is defined as the formation energies of two charge states are equal with setting the position of the Fermi-level in the fundamental band gap of hBN. The calculated charge transition levels for the considered defects are shown in Supplementary Figure 1. Carbon defects may be created by kinetic processes in experiments. One may define the binding energy of carbon defects to form larger carbon clusters as

$$E_{binding} = (\mathrm{C}_2)_n - (n\mathrm{C}_\mathrm{B} + n\mathrm{C}_\mathrm{N}) \tag{2}$$

, where n is the number C_2 units in the cluster, i.e., it is a $n = 1$ for the dimer and $n = 3$ for the 6C ring. The largest is the binding energy the more probable is that the defect will agglomerate if the defect species is able to diffuse. We find that 6C ring defect has the largest binding energy among the considered carbon clusters, although, in terms per C_2 unit, as shown in Supplementary Table 1.

For the excited state calculations with post-DFT method, the 6C defect was incorporated into a flake of hBN, containing 27 boron and 27 nitrogen atoms. The dangling bonds were passivated by hydrogen atoms. The calculations with the second-order approximate coupled cluster singles and doubles model (CC2)[10] and the algebraic diagrammatic construction method, ADC(2)[11] were performed with Turbomole code.[12,13]

The results of time-dependent (TD) DFT and n-electron valence state perturbation theory, NEVPT2(4,4)[14] were obtained by ORCA code.[15] In all calculations, we used cc-pVDZ basis set[16] and considered the PBE0 density functional[17] for TDDFT. To compute the HR factor by CC2, we first relaxed the system in both ground and excited states. Then, all atoms but hydrogen atoms were incorporated into the periodic lattice of hBN preserving their equilibrium positions. In this way, we could reach a consistent description of the sideband with the ΔSCF method, thereby relying on the PBE normal modes in both cases.

The periodic TD-PBE0 calculations were performed for the 6C defect, incorporated into

7x7 supercell of hBN by Quantum Espresso.[18] To this end, we employed Optimized Norm-Conserving Vanderbilt (ONCV) pseudopotentials [19] with the cutoff energy of 680 eV, and used the Davidson method for solving the Casida equation.[20] To break the periodicity along the out-of-plain direction, we introduced 1.25 nm of vacuum and used a truncation scheme for the Coulomb potential.[21]

Table 1: The calculated binding energy for clusters with even number of carbon atoms.

	C_NC_B	$4C_{pair}$	$4C_{chain}$	6C
Binding energy (eV)	-3.90	-7.43	-8.66	-14.88
Binding energy per C_2 unit (eV)	-3.90	-3.72	-4.33	-4.96

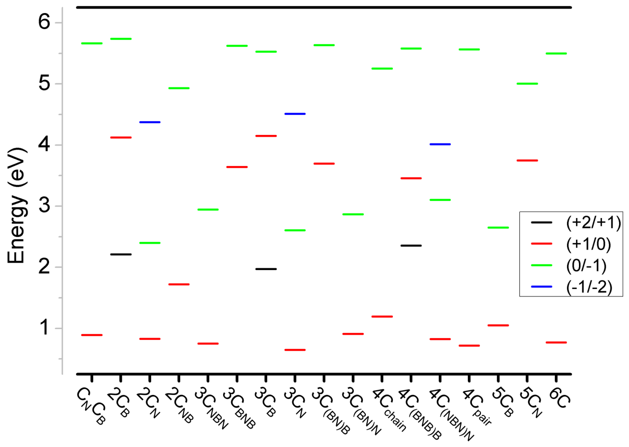

Figure 1: The charge transition levels for the defects considered.

Since the experimental PL signal features a short radiative lifetime, we only focus on those arrangements where the carbon atoms are closely packed within a single honeycomb. This structure serves as a basic and pivotal unit in the honeycomb structure and larger defects can be regarded as a combination of such. Noteworthy, the delocalization of defect orbitals should naturally decrease the excitation energy; therefore, larger defect complexes were not considered. In Supplementary Figure 2, we show the larger 10C structure while the energy difference between occupied and unoccupied defect states is small and the calculated ZPL could not match the experimental data. Larger defect complexes would further narrow the transition energy and they cannot explain the origin of 4.1 eV-emission.

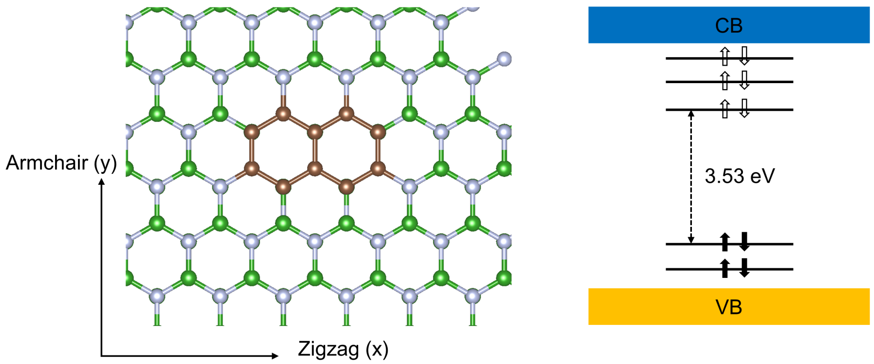

Figure 2: The geometry and electronic structure of 10C carbon cluster defect at ground state.

SUPPLEMENTARY NOTE 2: The two-hole wavefunctions with D_{3h} symmetry

The representations of the defect orbitals in the gap could be identified through their behaviour under inversion symmetry. Based on the wavefuntions plotted in Fig. 1(b) main text, we can see the sphere of four occupied states changes sign with horizontal reflection operator. Therefore, the states are labeled with e''. As mentioned in the main text, the excited states could be represented by two-hole wave funtions with determinants $|e''_{ox}e''_{ux}\rangle$, $|e''_{ox}e''_{uy}\rangle$, $|e''_{oy}e''_{ux}\rangle$, and $|e''_{oy}e''_{uy}\rangle$, where the subscript o and u indicate the occupied and unoccupied states, respectively. The wave functions then can be expressed for singlet and triplet as

$$\left.\begin{aligned}
|^1A'_1\rangle &= \mathcal{S}\tfrac{1}{\sqrt{2}}\left(|e''_{ox}e''_{ux}\rangle + |e''_{oy}e''_{uy}\rangle\right)\\
|^1E'_x\rangle &= \mathcal{S}\tfrac{1}{\sqrt{2}}\left(|e''_{ox}e''_{ux}\rangle - |e''_{oy}e''_{uy}\rangle\right)\\
|^1E'_y\rangle &= \mathcal{S}\tfrac{1}{\sqrt{2}}\left(|e''_{ox}e''_{uy}\rangle + |e''_{oy}e''_{ux}\rangle\right)\\
|^1A'_2\rangle &= \mathcal{S}\tfrac{1}{\sqrt{2}}\left(|e''_{ox}e''_{uy}\rangle - |e''_{oy}e''_{ux}\rangle\right)
\end{aligned}\right\}\otimes\mathcal{A}|\uparrow\downarrow\rangle$$

Table 2: The character table for D_{3h} point group.

	E	$2C_3$	$3C_2'$	σ_h	$2S_3$	$3\sigma_v$	linear,rotations	quadratic
A_1'	1	1	1	1	1	1		x^2+y^2, z^2
A_2'	1	1	-1	1	1	-1	R_z	
E'	2	-1	0	2	-1	0	(x,y)	x^2-y^2, xy
A_1''	1	1	1	-1	-1	-1		
A_2''	1	1	-1	-1	-1	1	z	
E''	2	-1	0	-2	1	0	(R_x, R_y)	xz, yz

$$\left.\begin{array}{l} |^3A_1'\rangle = \mathcal{A}\frac{1}{\sqrt{2}}\left(|e''_{ox}e''_{ux}\rangle + |e''_{oy}e''_{uy}\rangle\right) \\ |^3E_x'\rangle = \mathcal{A}\frac{1}{\sqrt{2}}\left(|e''_{ox}e''_{ux}\rangle - |e''_{oy}e''_{uy}\rangle\right) \\ |^3E_y'\rangle = \mathcal{A}\frac{1}{\sqrt{2}}\left(|e''_{ox}e''_{uy}\rangle + |e''_{oy}e''_{ux}\rangle\right) \\ |^3A_2'\rangle = \mathcal{A}\frac{1}{\sqrt{2}}\left(|e''_{ox}e''_{uy}\rangle - |e''_{oy}e''_{ux}\rangle\right) \end{array}\right\} \otimes \left\{\begin{array}{c} |\uparrow\uparrow\rangle \\ \mathcal{S}|\uparrow\downarrow\rangle \\ |\downarrow\downarrow\rangle \end{array}\right.$$

, where the first right-hand side term refers to the orbital part and the second one is for spin part (the arrows indicate the spin directions). Here, we use the antisymmetrization operator, $\mathcal{A}|xy\rangle = \frac{1}{\sqrt{2}}\left(|xy\rangle - |yx\rangle\right)$ for the singlet wavefunctions, and the symmetrization operator, $\mathcal{S}|xy\rangle = \frac{1}{\sqrt{2}}\left(|xy\rangle + |yx\rangle\right)$, for the triplets. nd we prove the equations according to the horizontal (σ_h) and (σ_v) reflection symmetry operators. We should note for the single particle of $|e''_x\rangle$, and $|e''_y\rangle$,

$$\sigma_h|e''_x\rangle = -|e''_x\rangle, \sigma_h|e''_y\rangle = -|e''_y\rangle$$

$$\sigma_v|e''_x\rangle = |e''_x\rangle, \sigma_v|e''_y\rangle = -|e''_y\rangle$$

Under σ_h symmetry, double prime representation should be excluded because the signs are kept as described as

$$\sigma_h \left\{\begin{array}{c} |A_1'\rangle \\ |E_x'\rangle \\ |E_y'\rangle \\ |A_2'\rangle \end{array}\right\} = \sigma_h(\mathcal{S}\ or\ \mathcal{A})\frac{1}{\sqrt{2}} \left\{\begin{array}{c} |e''_{ox}e''_{ux}\rangle + |e''_{oy}e''_{uy}\rangle \\ |e''_{ox}e''_{ux}\rangle - |e''_{oy}e''_{uy}\rangle \\ |e''_{ox}e''_{uy}\rangle + |e''_{oy}e''_{ux}\rangle \\ |e''_{ox}e''_{uy}\rangle - |e''_{oy}e''_{ux}\rangle \end{array}\right\} \otimes \text{spin} =$$

$$(\mathcal{S}\ or\ \mathcal{A})\frac{1}{\sqrt{2}}\left\{\begin{array}{c}|e''_{ox}e''_{ux}\rangle+|e''_{oy}e''_{uy}\rangle\\|e''_{ox}e''_{ux}\rangle-|e''_{oy}e''_{uy}\rangle\\|e''_{ox}e''_{uy}\rangle+|e''_{oy}e''_{ux}\rangle\\|e''_{ox}e''_{uy}\rangle-|e''_{oy}e''_{ux}\rangle\end{array}\right\}\otimes\text{spin}=\left\{\begin{array}{c}|A'_1\rangle\\|E'_x\rangle\\|E'_y\rangle\\|A'_2\rangle\end{array}\right\}$$

Under σ_v symmetry operation,

$$\sigma_v\left\{\begin{array}{c}|A'_1\rangle\\|E'_x\rangle\\|E'_y\rangle\\|A'_2\rangle\end{array}\right\}=\sigma_v(\mathcal{S}\ or\ \mathcal{A})\frac{1}{\sqrt{2}}\left\{\begin{array}{c}|e''_{ox}e''_{ux}\rangle+|e''_{oy}e''_{uy}\rangle\\|e''_{ox}e''_{ux}\rangle-|e''_{oy}e''_{uy}\rangle\\|e''_{ox}e''_{uy}\rangle+|e''_{oy}e''_{ux}\rangle\\|e''_{ox}e''_{uy}\rangle-|e''_{oy}e''_{ux}\rangle\end{array}\right\}\otimes\text{spin}=$$

$$(\mathcal{S}\ or\ \mathcal{A})\frac{1}{\sqrt{2}}\left\{\begin{array}{c}|e''_{ox}e''_{ux}\rangle+|e''_{oy}e''_{uy}\rangle\\|e''_{ox}e''_{ux}\rangle-|e''_{oy}e''_{uy}\rangle\\-|e''_{ox}e''_{uy}\rangle-|e''_{oy}e''_{ux}\rangle\\-|e''_{ox}e''_{uy}\rangle+|e''_{oy}e''_{ux}\rangle\end{array}\right\}\otimes\text{spin}=\left\{\begin{array}{c}|A'_1\rangle\\|E'_x\rangle\\-|E'_y\rangle\\-|A'_2\rangle\end{array}\right\}$$

For simplicity, we further simplify the equations by omitting the symmetry operator and spin part of the wave functions as

$$\left\{\begin{array}{c}|A'_1\rangle\\|E'_x\rangle\\|E'_y\rangle\\|A'_2\rangle\end{array}\right\}=\frac{1}{\sqrt{2}}\begin{pmatrix}1&1&0&0\\1&-1&0&0\\0&0&1&1\\0&0&1&-1\end{pmatrix}\begin{pmatrix}|e''_xe''_x\rangle\\|e''_ye''_y\rangle\\|e''_xe''_y\rangle\\|e''_ye''_x\rangle\end{pmatrix}=\frac{1}{\sqrt{2}}\begin{pmatrix}1&1&0&0\\1&-1&0&0\\0&0&1&1\\0&0&1&-1\end{pmatrix}\begin{pmatrix}|xx\rangle\\|yy\rangle\\|xy\rangle\\|yx\rangle\end{pmatrix}\tag{3}$$

$$\begin{Bmatrix} |A_2'\rangle \\ |E_y'\rangle \\ |E_x'\rangle \\ |A_1'\rangle \end{Bmatrix} = \frac{1}{\sqrt{2}} \begin{pmatrix} 1 & -1 & 0 & 0 \\ 1 & 1 & 0 & 0 \\ 0 & 0 & 1 & -1 \\ 0 & 0 & 1 & 1 \end{pmatrix} \begin{pmatrix} |e_x''e_y''\rangle \\ |e_y''e_x''\rangle \\ |e_x''e_x''\rangle \\ |e_y''e_y''\rangle \end{pmatrix} = \frac{1}{\sqrt{2}} \begin{pmatrix} 1 & -1 & 0 & 0 \\ 1 & 1 & 0 & 0 \\ 0 & 0 & 1 & -1 \\ 0 & 0 & 1 & 1 \end{pmatrix} \begin{pmatrix} |xy\rangle \\ |yx\rangle \\ |xx\rangle \\ |yy\rangle \end{pmatrix} \tag{4}$$

which can be transformed to

$$\begin{pmatrix} |xy\rangle \\ |yx\rangle \\ |xx\rangle \\ |yy\rangle \end{pmatrix} = \frac{1}{\sqrt{2}} \begin{pmatrix} 1 & 1 & 0 & 0 \\ -1 & 1 & 0 & 0 \\ 0 & 0 & 1 & 1 \\ 0 & 0 & -1 & 1 \end{pmatrix} \begin{Bmatrix} |A_2'\rangle \\ |E_y'\rangle \\ |E_x'\rangle \\ |A_1'\rangle \end{Bmatrix} \tag{5}$$

SUPPLEMENTARY NOTE 3: The electronic term $\hat{W}$ Hamiltonian

We could apply these on the electronic correlation term $\hat{W}$.

$$\begin{aligned}
&\hat{W} = \Lambda(|A_1'\rangle\langle A_1'| - |A_2'\rangle\langle A_2'|) - \Delta(|E_x'\rangle\langle E_x'| + |E_y'\rangle\langle E_y'|), \\
&\langle xx|\hat{W}|xx\rangle = \frac{1}{2}(\langle A_1'|\hat{W}|A_1'\rangle + \langle E_x'|\hat{W}|E_x'\rangle + \langle A_1'|\hat{W}|E_x'\rangle + \langle E_x'|\hat{W}|A_1'\rangle) = \frac{1}{2}\Lambda - \frac{1}{2}\Delta, \\
&\langle yy|\hat{W}|yy\rangle = \frac{1}{2}(\langle A_1'|\hat{W}|A_1'\rangle - \langle E_x'|\hat{W}|E_x'\rangle + \langle A_1'|\hat{W}|E_x'\rangle - \langle E_x'|\hat{W}|A_1'\rangle) = \frac{1}{2}\Lambda + \frac{1}{2}\Delta, \\
&\langle xy|\hat{W}|xy\rangle = \frac{1}{2}(\langle A_2'|\hat{W}|A_2'\rangle + \langle E_y'|\hat{W}|E_y'\rangle + \langle A_2'|\hat{W}|E_y'\rangle + \langle E_y'|\hat{W}|A_2'\rangle) = -\frac{1}{2}\Lambda - \frac{1}{2}\Delta, \\
&\langle yx|\hat{W}|yx\rangle = \frac{1}{2}(-\langle A_2'|\hat{W}|A_2'\rangle + \langle E_y'|\hat{W}|E_y'\rangle + \langle A_2'|\hat{W}|E_y'\rangle - \langle E_y'|\hat{W}|A_2'\rangle) = \frac{1}{2}\Lambda - \frac{1}{2}\Delta.
\end{aligned} \tag{6}$$

One could see that $\langle xx|\hat{W}|xx\rangle = \langle yx|\hat{W}|yx\rangle$, and these two are the E' states. $\langle yy|\hat{W}|yy\rangle = -\langle xy|\hat{W}|xy\rangle$, these belong to A' states. According to the Λ and Δ values we get, it is possible to energetically order the four states. Here, we define $\Lambda > 0$ in singlet while $\Lambda < 0$ in triplet,

and $\Delta < 0$. For singlet $\langle yy|\hat{W}|yy\rangle > \langle xy|\hat{W}|xy\rangle$, and the lowest adibatic potential energy surface (APES) layer should demonstrate partial A_2' while for triplet the lowest one contains A_1' state because $\langle yy|\hat{W}|yy\rangle < \langle xy|\hat{W}|xy\rangle$. Based on above expression, we can have matrix form of the $\hat{W}$[22,23] as

$$\hat{W} = \frac{1}{2}\Lambda \underbrace{\begin{bmatrix} 1 & 1 & 0 & 0 \\ 1 & 1 & 0 & 0 \\ 0 & 0 & 0 & 0 \\ 0 & 0 & 0 & 0 \end{bmatrix}}_{|A_1'\rangle\langle A_1'|} - \frac{1}{2}\Delta \underbrace{\begin{bmatrix} 1 & -1 & 0 & 0 \\ -1 & 1 & 0 & 0 \\ 0 & 0 & 0 & 0 \\ 0 & 0 & 0 & 0 \end{bmatrix}}_{|E_x'\rangle\langle E_x'|} - \frac{1}{2}\Delta \underbrace{\begin{bmatrix} 0 & 0 & 0 & 0 \\ 0 & 0 & 0 & 0 \\ 0 & 0 & 1 & 1 \\ 0 & 0 & 1 & 1 \end{bmatrix}}_{|E_y'\rangle\langle E_y'|} - \frac{1}{2}\Lambda \underbrace{\begin{bmatrix} 0 & 0 & 0 & 0 \\ 0 & 0 & 0 & 0 \\ 0 & 0 & 1 & -1 \\ 0 & 0 & -1 & 1 \end{bmatrix}}_{|A_2'\rangle\langle A_2'|}$$
$$= \frac{1}{2}\Lambda \begin{bmatrix} 1 & 1 & 0 & 0 \\ 1 & 1 & 0 & 0 \\ 0 & 0 & -1 & 1 \\ 0 & 0 & 1 & -1 \end{bmatrix} - \frac{1}{2}\Delta \begin{bmatrix} 1 & -1 & 0 & 0 \\ -1 & 1 & 0 & 0 \\ 0 & 0 & 1 & 1 \\ 0 & 0 & 1 & 1 \end{bmatrix} \tag{7}$$

As shown in main text, the energy difference between the two configurations, computed by ΔSCF, are 41 meV and 308 meV for singlets and triplets, respectively. From the CC2 results, we have computed Λ and Δ of -168.5 and -619.5 meV for the singlets and of 393 meV and 7 meV for the triplets, respectively.In Table 3 and 4, we list the excited states calculated by different methods with flake (periodic) model. The results show similar trend that, in the singlet manifold, two non-degenerate states have the lowest energies while the degenerate state lies between the two non-degenerate ones in the triplet manifold. Another pronounced feature of TDDFT to be mentioned, is that it severely overestimates the energy gap between the lowest singlet and triplet states. Of note, the latter behaviour is largely reminiscent on the performance of this approach for the multiresonant organic emitters.[24]

SUPPLEMENTARY NOTE 4: Derivation of the product Jahn-Teller Hamiltonian

$$\hat{H}_{\mathrm{JT}} = F_o \left(\hat{\sigma}_z \otimes \hat{\sigma}_0 \hat{x} + \hat{\sigma}_x \otimes \hat{\sigma}_0 \hat{y} \right) + F_u \left(\hat{\sigma}_0 \otimes \hat{\sigma}_z \hat{x} + \hat{\sigma}_0 \otimes \hat{\sigma}_x \hat{y} \right), \tag{8}$$

. With the 2×2 Pauli matrices:

$$\hat{\sigma}_z - \hat{\sigma}_0 = \begin{bmatrix} 1 & 0 \\ 0 & -1 \end{bmatrix} \otimes \begin{bmatrix} 1 & 0 \\ 0 & 1 \end{bmatrix} = \begin{bmatrix} 1 & 0 & 0 & 0 \\ 0 & -1 & 0 & 0 \\ 0 & 0 & 1 & 0 \\ 0 & 0 & 0 & -1 \end{bmatrix} \tag{9}$$

$$\hat{\sigma}_x - \hat{\sigma}_0 = \begin{bmatrix} 0 & 1 \\ 1 & 0 \end{bmatrix} \otimes \begin{bmatrix} 1 & 0 \\ 0 & 1 \end{bmatrix} = \begin{bmatrix} 0 & 1 & 0 & 0 \\ 1 & 0 & 0 & 0 \\ 0 & 0 & 0 & 1 \\ 0 & 0 & 1 & 0 \end{bmatrix}, \tag{10}$$

$$\hat{\sigma}_0 - \hat{\sigma}_z = \begin{bmatrix} 1 & 0 \\ 0 & 1 \end{bmatrix} \otimes \begin{bmatrix} 1 & 0 \\ 0 & -1 \end{bmatrix} = \begin{bmatrix} 1 & 0 & 0 & 0 \\ 0 & 1 & 0 & 0 \\ 0 & 0 & -1 & 0 \\ 0 & 0 & 0 & -1 \end{bmatrix} \tag{11}$$

$$\hat{\sigma}_0 - \hat{\sigma}_x = \begin{bmatrix} 1 & 0 \\ 0 & 1 \end{bmatrix} \otimes \begin{bmatrix} 0 & 1 \\ 1 & 0 \end{bmatrix} = \begin{bmatrix} 0 & 0 & 1 & 0 \\ 0 & 0 & 0 & 1 \\ 1 & 0 & 0 & 0 \\ 0 & 1 & 0 & 0 \end{bmatrix}, \tag{12}$$

we could have the linear vibronic Hamiltonian in Eq. 8 given as

$$\underbrace{\begin{bmatrix} \hat{x}(F_o+F_u) & \hat{y}F_o & \hat{y}F_u & 0 \\ \hat{y}F_o & -\hat{x}(F_o-F_u) & 0 & \hat{y}F_u \\ \hat{y}F_u & 0 & \hat{x}(F_o-F_u) & \hat{y}F_o \\ 0 & \hat{y}F_u & \hat{y}F_o & -\hat{x}(F_o+F_u) \end{bmatrix}}_{|e''_{ox}e''_{ux}\rangle \quad |e''_{ox}e''_{uy}\rangle \quad |e''_{oy}e''_{ux}\rangle \quad |e''_{oy}e''_{uy}\rangle}, \tag{13}$$

where the diagonal part of this expression indicates that with $\pm\hat{x}$ displacement, the energy of single determinants change their energies with constructive and destructive joint vibronic coupling strength $F_o \pm F_u$; $\hat{H}_{\mathrm{JT}}$ is a iso-stationary function for the APES of the JT system.

Based on these data, the electron-phonon coupling parameters are calculated as,

$$E^1_{\mathrm{JT}} = \frac{(F_o+F_u)^2}{2\hbar\omega_E}, E^2_{\mathrm{JT}} = \frac{(F_o-F_u)^2}{2\hbar\omega_E}. \tag{14}$$

SUPPLEMENTARY NOTE 5: The four layers product Jahn-Teller system and the lowest polaronic state

In the double degenerate Jahn-Teller (JT) system, the vibronic ground states in each E branch can be written as

$$|\tilde{E}^\Psi_x\rangle = |\tilde{\Psi}_{x,R,\varphi}\rangle = \cos\frac{\varphi}{2}|e_x\rangle - \sin\frac{\varphi}{2}|e_y\rangle, |\tilde{E}^\Psi_y\rangle = |\tilde{\Psi}_{y,R,\varphi}\rangle = \sin\frac{\varphi}{2}|e_x\rangle + \cos\frac{\varphi}{2}|e_y\rangle, \tag{15}$$

therefore the four single determinants can be expressed by

$$\begin{aligned} |e''_x e''_x\rangle^\Psi &= \tfrac{1}{2}[|xx\rangle + |yy\rangle + \cos\varphi(|xx\rangle - |yy\rangle) - \sin\varphi(|xy\rangle + |yx\rangle)] = \tfrac{1}{\sqrt{2}}[|A'_1\rangle + \cos\varphi|E'_x\rangle - \sin\varphi|E'_y\rangle] \\ |e''_y e''_y\rangle^\Psi &= \tfrac{1}{2}[|yy\rangle + |xx\rangle + \cos\varphi(|yy\rangle - |xx\rangle) + \sin\varphi(|yx\rangle - |xy\rangle)] = \tfrac{1}{\sqrt{2}}[|A'_1\rangle - \cos\varphi|E'_x\rangle + \sin\varphi|E'_y\rangle] \\ |e''_x e''_y\rangle^\Psi &= \tfrac{1}{2}[|xy\rangle - |yx\rangle + \cos\varphi(|xy\rangle + |yx\rangle) + \sin\varphi(|xx\rangle - |yy\rangle)] = \tfrac{1}{\sqrt{2}}[|A'_2\rangle + \cos\varphi|E'_y\rangle - \sin\varphi|E'_x\rangle] \\ |e''_y e''_x\rangle^\Psi &= \tfrac{1}{2}[|yx\rangle - |xy\rangle + \cos\varphi(|yx\rangle + |xy\rangle) + \sin\varphi(|xx\rangle - |yy\rangle)] = \tfrac{1}{\sqrt{2}}[-|A'_2\rangle + \cos\varphi|E'_y\rangle - \sin\varphi|E'_x\rangle] \end{aligned}$$

To calculate the lowest APES, a phase factor should be added to Eq. 15 to change the sign of wave function with full rotation along the bottom of the APES. The vibronic ground state could be expressed by[25]

$$
\begin{aligned}
e^{\pm i\varphi}|\tilde{\Psi}_{R,\varphi}\rangle &= e^{\pm i\varphi}[\cos\frac{\varphi}{2}|E_x\rangle - \sin\frac{\varphi}{2}|E_y\rangle], or \\
e^{\pm i\varphi}|\tilde{\Psi}_{R,\varphi}\rangle &= e^{\pm i\varphi}[\sin\frac{\varphi}{2}|E_x\rangle + \cos\frac{\varphi}{2}|E_y\rangle].
\end{aligned}
\tag{16}
$$

The first one is for the lower layer while the second one is for the upper layer. Here we just focus on the ground state therefore the first one is considered. Then the effect of double degenerate localized mode $\tilde{E}''_o \otimes \tilde{E}''_u$ have same transformation of $|e'_y e''_y\rangle^\Psi$ or $|e'_x e''_x\rangle^\Psi$ as

$$
\begin{aligned}
&|\tilde{E}''_o\rangle \otimes |\tilde{E}''_u\rangle = \\
&\frac{1}{\sqrt{2}}\begin{Bmatrix} 1 \\ e^{-2i\varphi} \\ e^{+2i\varphi} \end{Bmatrix}[|A'_1\rangle \pm \cos(\varphi)|E'_x\rangle \mp \sin(\varphi)|E'_y\rangle].
\end{aligned}
\tag{17}
$$

This indicates that the minima loop solely constitutes of mixed states of $|A'_1\rangle$ and $|E'\rangle$. The polaronic wave function with this minima loop with full rotation included can be solved by

$$
\begin{aligned}
|\tilde{\Phi}\rangle = &\sum_{n,m}[a_{n,m}|e''_{ox}e''_{ux}\rangle + b_{n,m}|e''_{ox}e''_{uy}\rangle \\
&+ c_{n,m}|e''_{oy}e''_{ux}\rangle + d_{n,m}|e''_{oy}e''_{uy}\rangle] \otimes |n,m\rangle,
\end{aligned}
\tag{18}
$$

where we consider the expansion within 40 oscillator quanta $(n+m)$ for the coefficient parameters. We could see that for both the singlet and triplet, the JT distortion is irrelevant to the A'_2 state, as shown in Fig. 3 of the main text that the A'_2 does not have JT instability. As the symmetry breaking, the irreducible representations change their character as shown in Table 5. The E' would partially contribute to both A' states while majorly to A'_1 considering the JT mixture, as well as in the oscillator strengths shown in Table 6.

Table 3: The excited states calculated by different methods with flake model. The energy unit is eV.

TD-PBE0		NEVPT2		SOS-ADC2		CC2	
Singlet	Triplet	Singlet	Triplet	Singlet	Triplet	Singlet	Triplet
4.303	3.600	3.865	3.677	4.241	4.022	4.255	3.919
4.640	3.936	4.754	3.969	4.891	4.399	4.765	4.305
5.091	3.936	4.900	3.969	5.365	4.399	5.122	4.305
5.091	4.120	4.900	4.627	5.365	4.958	5.123	4.704

Table 4: The singlet excited states calculated from TD-PBE0 with different models. The energy unit is eV.

Periodic	Flake	Energy difference
4.266	4.303	0.037
4.522	4.640	0.118
4.793	5.091	0.298

Table 5: Subgroup correlation table of the D_{3h} point group. The columns show the effect of symmetry lowering on the high symmetry irreducible representations.

group	irreducible representations		
D_{3h}	A_1'	A_2'	E'
$C_{2v}(xy)$	A_1	B_1	A_1+B_1

SUPPLEMENTARY NOTE 6: Intersystem crossing and the non-radiative decay from $|A_1'\rangle$ to $|A_2'\rangle$

Beside the radiative decay, we have also explored a possibility of the non-radiative transition to the triplet manifold through the intersystem crossing (ISC). This process is mainly governed by the spin-orbit coupling (SOC), and the possible pathways are depicted in Fig. 4(d) in main text. The SOC interaction can be expressed as

$$\hat{H}_{\text{SO}} = \sum_k \lambda_{x,y}(l_k^x s_k^x + l_k^y s_k^y) + \lambda_z(l_k^z s_k^z + l_k^z s_k^z), \tag{19}$$

where $\lambda_{x,y}$ are the non-axial components, while λ_z is an axial component. In particular, $\lambda_{x,y}$ couples the triplet states with the non-zero spin projections ($m_s = \pm1$) with singlets of

Table 6: The calculated oscillator strengths in atomic unit with TD-PBE0 for the configurations with D_{3h} and C_{2v} symmetry.

	D_{3h}	C_{2v}
$\lvert A_2'\rangle$	0.000000029	0.001197691
$\lvert A_1'\rangle$	0.000021498	0.166151140
$\lvert E_x'\rangle$	0.936052340	0.896887289
$\lvert E_y'\rangle$	0.937635100	0.740672794

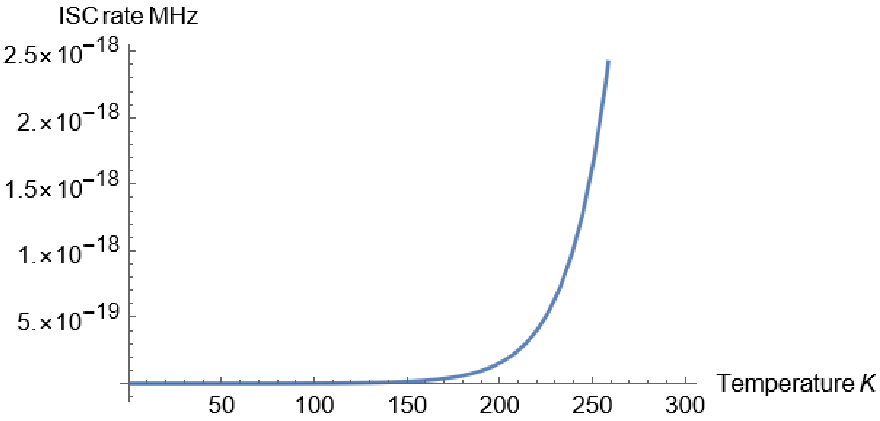

Figure 3: The temperature dependent ISC rate from singlet to triplet.

different electronic configuration. In turn, λ_z links states with $m_s = 0$ spin projections with the states of the same electronic configuration. Since all the excited states in our system have the same electronic configuration $|e''e''\rangle$ only the axial part is non-vanishing. The SOC splits ${}^3E'$ into $m_s = \pm 1$ sub-states $A_{1,2}$ and $E_{1,2}$ with the $m_s = 0$ $E_{x,y}$ state. In addition, ${}^1A_1'$ could also decay to ${}^3E'$ due to the mixture with ${}^1E'$. The ISC rate from singlet to triplet can be calculated by[26]

$$\tau_{\text{ISC}} = 4\pi\hbar\lambda_z^2 F(\Delta E), \tag{20}$$

where F is the spectral function of vibrational overlap between the singlets and triplet states, and ΔE is the energy splitting between singlet and triplet levels. From the TDDFT calculations, we found the largest value of λ_z of only 1.5 GHz. Given a considerable energy gap between the states, this translates into the enormously large τ_{ISC}, and disables the ISC process in the zeroth-order (see Supplementary Figure 3). Yet, we note that the triplet

manifold may be populated via a nongeminate recombination of hot charge carriers achieved by a two-photon absorption process. Another important issue is the non-radative decay from the JT distorted $|A_1'\rangle$ state to the low lying dark $|A_2'\rangle$ state. The defect would be a dim center if the non-radiative decay is faster than the radiative process and would bleach itself. The non-radiative transition rate (Γ_{nrad}) between the two states can be calculated[27] as

$$\Gamma_{\mathrm{nrad}} = \frac{2\pi}{\hbar} g W_{if}^2 X_{if}(T),$$
$$X_{if}(T) = \sum_{n,m} p_{in} |\chi_i|\hat{Q} - Q_0|\chi_f|^2 \times \delta(n\hbar\omega_i - m\hbar\omega_f + \Delta E_{if}),$$
$$W_{if} = \langle\psi_i|\partial_Q \hat{H}|\psi_f\rangle.$$

Here, W_{if} is the electronic term and $X_{if}(T)$ is temperature dependent phonon term. g is the equivalent energy-degenerate atomic configurations and p_{in} is the thermal population. i and f correspond the initial and final state and here the C_{2v} and D_{3h} are used to generate the configuration coordinates. The phonon matrix $|\chi_i|\hat{Q} - Q_0|\chi_f|$ sums up the harmonic oscillator wavefunctions that enters the non-radiative recombination process. ΔE_{if} is energy difference between the two states and ψ is the single particle wavefunction from DFT. The electron-phonon coupling matrix can be mimic by the single particle wave function overlap between $|e''_{ox}\rangle$ and $|e''_{oy}\rangle$ together with $|e''_{ux}\rangle$ and $|e''_{uy}\rangle$, as shown in Supplementary Figure 4. $\hat{H}$ is the Kohn-Sham Hamiltonian and can be expressed as energy splitting between e state as a function of Q. The corresponding W_{if} is 0.00011 $eVamu^{-1/2-1}$ and non-radiative rate is 509 MHz.

The total transition rate from $|A_1'\rangle$ is

$$\frac{1}{\tau} = \frac{1}{\tau_{\mathrm{nrad}}} + \frac{1}{\tau_{\mathrm{rad}}}.$$

The averaged rate is 319 MHz. Also based on $\frac{\Gamma_{\mathrm{rad}}}{\Gamma}$, where Γ is the total transition rate, we can estimate the quantum yield is about 62.7%, however, this is the optimal value without

considering the temperature effect.

SUPPLEMENTARY NOTE 7: The temperature dependent quantum yield

Temperature influences the thermal distribution between the polaronic states $|\tilde{A}_1'\rangle$ and $|\tilde{E}'\rangle$ and further influence the brightness, i.e., PL intensity. This process τ_{23} is in picosecond range (0.17 ps at 100 K for singlet) and much faster than the non-radiative decay to low-lying $|A_2'\rangle$ according to $\hbar\omega_E e^{-\delta/k_BT}$.[28,29] At room temperature, 76% electrons occupy the bright $|\tilde{E}'\rangle$ state and this accounts for the radiative decay process. Here we evaluate the temperature effect according following equation,

$$\frac{\Gamma_{\text{rad}} e^{-\delta/k_BT}}{\Gamma_{\text{rad}} e^{-\delta/k_BT} + \Gamma_{\text{nrad}} e^{-\delta/k_BT}},$$

where δ is given in the main text. The k_B is the Boltzmann constant. For the non-radiative part, the temperature effect is plotted in Supplementary Figure 4c. We assume the non-radiative decay rate remains the same for the two polaronic states $\Gamma_{31} \approx \Gamma_{21}$. The temperature dependent quantum yield is plotted in Supplementary Figure 5. At low temperature region the brightness is close to zero due to small radiative decay and it is about 52.1% at 300 K. There is non-radiative decay from $|A_2'\rangle$ to ground state, however, this is slow due to the large band gap. Therefore the $|A_2'\rangle$ could be a relative long-live excited metastable state and can be further excited to conduction band by two-photon excitation.

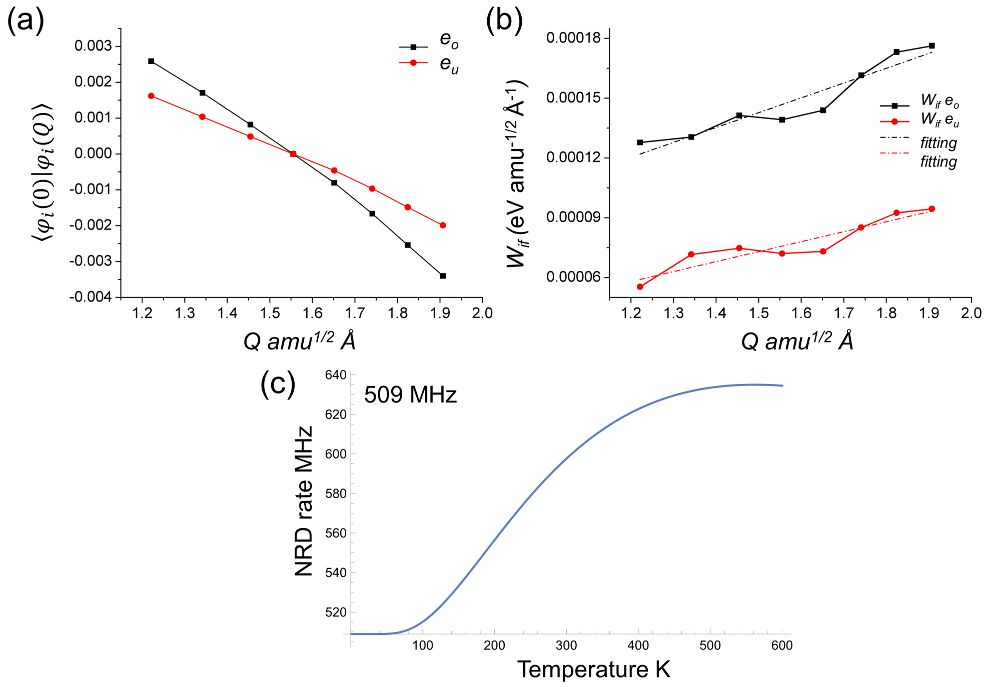

Figure 4: (a) The overlap between e state wavefunctions. (b) The electronic term W_{if} and linear fitting result. (c) The temperature dependent non-radiative transition rate from $|A_1'\rangle$ to $|A_2'\rangle$

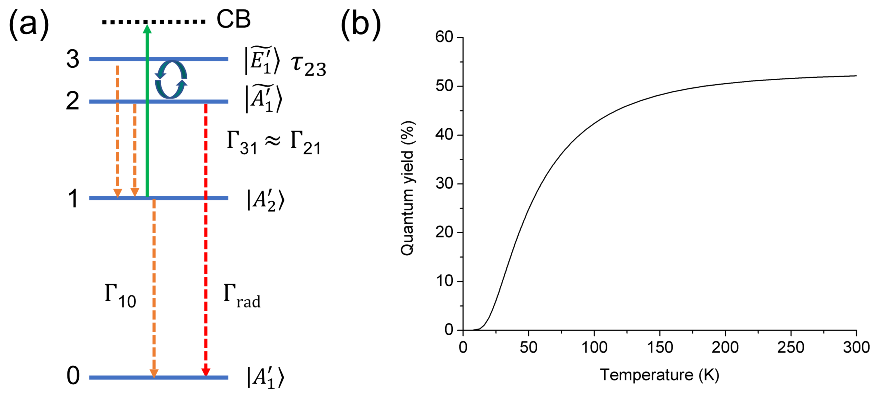

Figure 5: (a) The radiative and non-radiative transition. The green cycle denotes the thermal population between polaronic states $|\tilde{A}_1'\rangle$ to $|\tilde{E}'\rangle$. The time τ_{23} for this process is in picosecond range. Considering this we assume the non-radiative transitions from the polaronic states to low-lying dark $|\tilde{A}_2'\rangle$ are the same $\Gamma_{31} \approx \Gamma_{21}$. (b) The temperature dependent quantum yield.

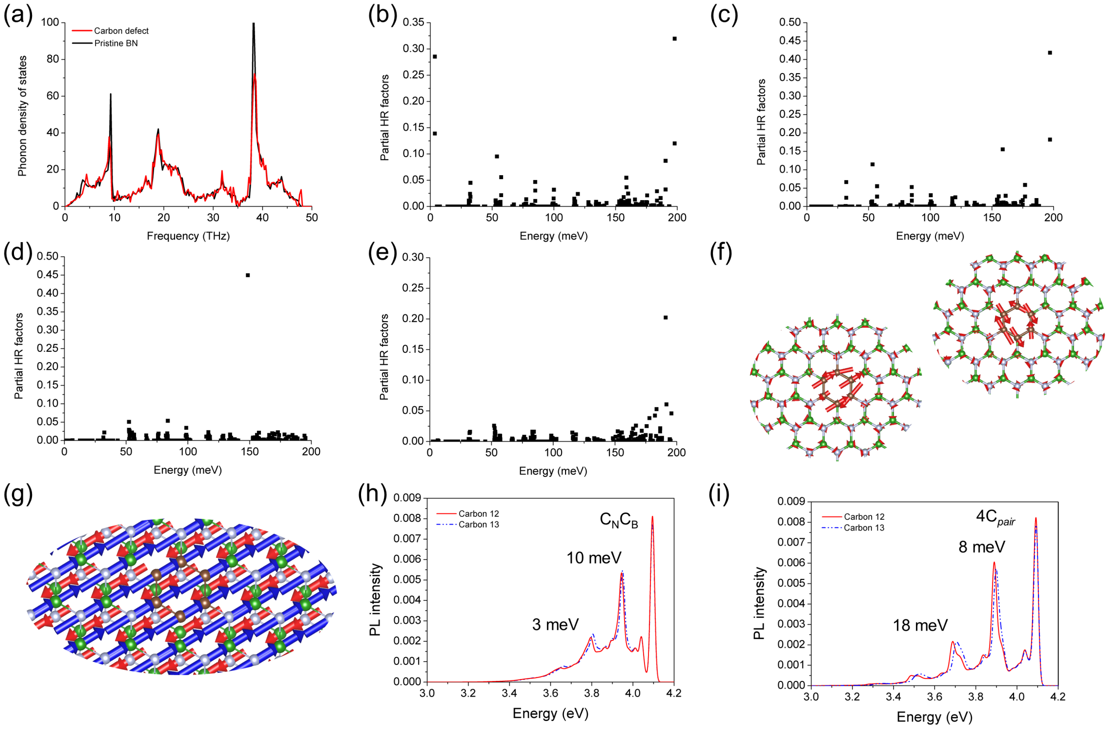

Figure 6: (a) The calculated phonon density of state (PDOS) of the pristine and 6C defective hBN. The most prominent peak appears at about 200 meV (≈50 THz), corresponding to the localized E phonon. (b) The partial HR factor of the 6C defect in bulk model. (c) The partial HR factor of the 6C defect in single layer model. (d) The partial HR factor of the C_NC_B in bulk model. (e) The partial HR factor of the $4C_{pair}$ in bulk model. (f) The visualization of the localized double degenerate E phonon modes. (g) The visualization of the degenerate E phonon modes at low energy. The top and bottom layer atoms move the opposite directions. (h) The simulated PL spectrum of dimer defect (C_NC_B). (i) The simulated PL spectrum of double dimer defect ($4C_{pair}$).

SUPPLEMENTARY NOTE 8: Phonon properties related to carbon defects

The calculated phonon densities of states for the perfect hBN and one with the ring 6C defect are shown in Supplementary Figure 6. The defect introduces new phonon modes with the most prominent one locating at 200 meV. This peak also has largest contribution for the partial HR factor. We also find two degenerate modes at low energy range (3 meV) which are the collective motion of atoms, as shown in (g). They represent a mutual displacement of the hBN layers and are naturally missing for the monolayer configuration. The two modes are related to the D_{3h} symmetry while they are missing at single layer model and C_NC_B and $4C_{pair}$. Without these two modes, the HR factor can reduce to 1.7. This phenomena motivated us to calculate the single layer case. As expected, we get $S = 1.8$ with the same parameters in bulk. However, for single layer model, the GW band gap is about 7.1 eV,[27] therefore, to reproduce it the mixing parameter α should be further increased to 0.5. Under this condition, the calculated ZPL for 6C ring defect is 5.02 eV and $S = 2.14$.

In particular, to confirm the involvement of carbon in a colour centre, it is commonplace to use the isotopic purification method, that incorporates ^{13}C into the lattice of the material during its growth. Here, we determine the isotopic shift in the emission energy and sideband for the 6C defect associated by replacing ^{12}C with ^{13}C isotope. First, we calculated the sideband with 100% of ^{13}C isotopes and found that the HR factor reduces to 1.78, see Figure 6(e) in main text. In turn, the phonon replicas show a blue shift by $\sim$10 meV. For comparison, the isotopic effect on C_NC_B introduces and the $4C_{pair}$ has similar blue shift but with different values as shown in Supplementary Figure 6. This might provide a feasible way to differentiate the configurations for emissions.

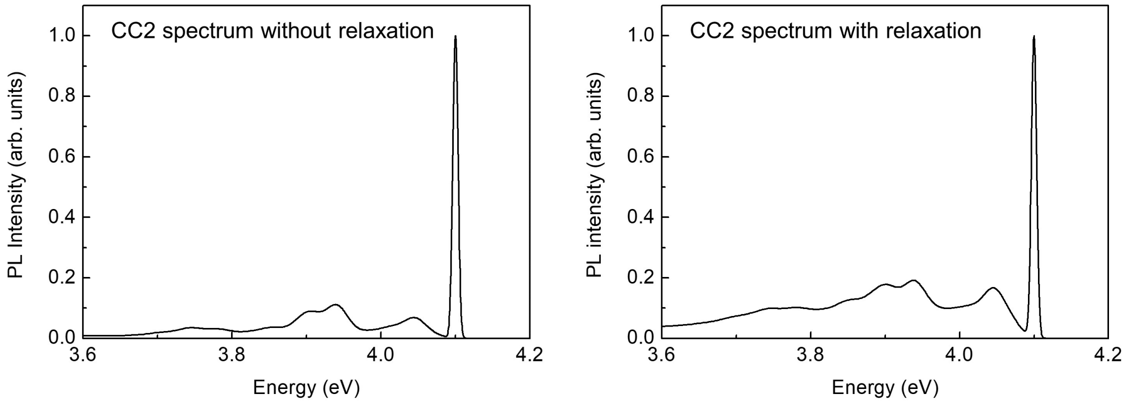

Figure 7: The simulated PL spectrum of 6C defect with and without environmental atoms relaxation. The cluster is relaxed with CC2 at ground and excited state and then embedded in periodic supercell. The rest environmental atoms are relaxed with DFT HSE functionals. The ZPL is shifted to experimental 4.1 eV.

SUPPLEMENTARY NOTE 9: Strain effect on C_NC_B and 6C

To evaluate the strain effect, we apply -2% to +2% strain along zigzag and armchair directions as shown in Supplementary Figure 8 which could be mapped out in experiment.[30] A $5 \times 3\sqrt{3}$ square supercell is used with C_NC_B and 6C considered and the calculation parameters are the same as mentioned in the Supplementary Note 1. With applied strain, the symmetry of carbon cluster (6C) reduces to C_{2v} so the pJT solution is not included.

For simplicity, here we just calculate the vertical excitation. For C_NC_B the strain is applied parallel (armchair) or perpendicular (zigzag) to the C-C dimer bond and the transition is between b_2 defect state so the excitation energy evolution demonstrates nearly linear dependence and decreases as increase of strain. Strain along zigzag direction shows smaller influence since it does not alter the C-C bond length while the strain along armchair does. In 6C, the situation is much more complex. Generally, strain decreases the excitation energy however it is not linear response. We speculate the strain might be an effective way to distinguish the type of carbon defects, at least for C_NC_B and 6C. Also, we have to admit the strain we consider is the simplest case while the shear strain is not included. The

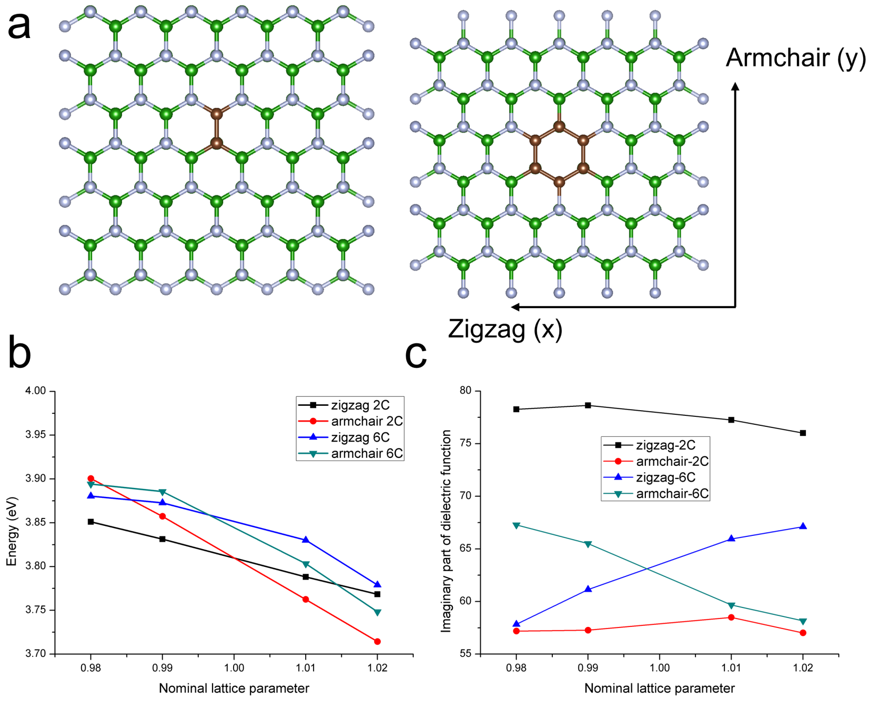

Figure 8: (a) The strain directions on C_NC_B and 6C. (b) The vertical excitation energy evolution and (c) imaginary part of dielectric function as function of external strain. X = 1 corresponds to the structure without strain which is not included.

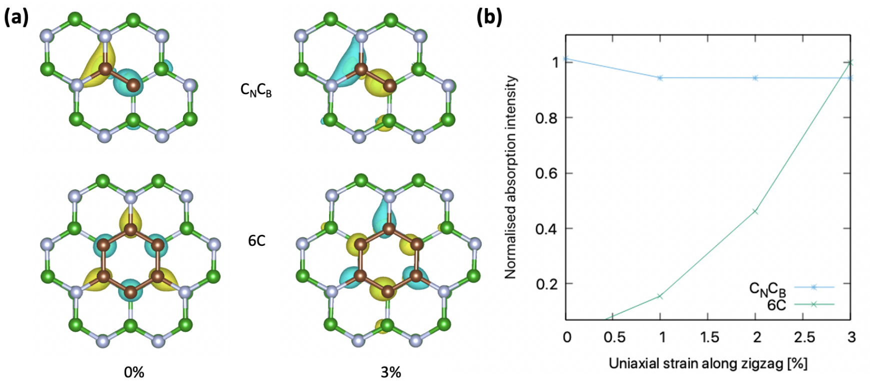

Figure 9: The effect on uniaxial density, applied along the zigzag direction, on (a) response density and (b) absorption intensity for the optically active transitions in C_NC_B and 6C defect.

transition dipole moment evolution can be deduced from the imaginary part of dielectric function. For C_NC_B the dipole moment does not change much. As increase of strain on 6C, the intensity of dipole moment increases along zigzag direction while decreases along armchair direction. The lifetime would change more dramatically for strain along armchair since both the transition energy and transition dipole moment decrease.

In particular, even more spectacular effect is expected for the intensity of the optical signal. To this end, we considered the effect of the applied strain on the absorption intensity by TD-PBE0, see Supplementary Figure 9. The calculations show that for C_NC_B, the uniaxial strain does not alter the response density. As a result, no significant evolution of the intensity is observed. By contrast, in a D_{3h} configuration, the efficient absorption in the A_1 state of 6C is hindered by the presence of C_3 symmetry axes. Upon the effect of strain along the zigzag direction, this symmetry constraint is lifted. The response density primary localises along the armchair direction, while the change in the resulting transition dipole moment from 0 to 3% of strain increases the absorption intensity by more than an order of magnitude.

References

(1) Kresse, G.; Furthmüller, J. efficiency of ab-initio total energy calculations for metals and semiconductors using a plane-wave basis set. *Comput. Mater. Sci.* **1996**, *6*, 15–50.

(2) Kresse, G.; Furthmüller, J. efficient iterative schemes for ab initio total-energy calculations using a plane-wave basis set. *Phys. Rev. B* **1996**, *54*, 11169.

(3) Blöchl, P. E. projector augmented-wave method. *Phys. Rev. B* **1994**, *50*, 17953.

(4) Kresse, G.; Joubert, D. from ultrasoft pseudopotentials to the projector augmented-wave method. *Phys. Rev. B* **1999**, *59*, 1758.

(5) Heyd, J.; Scuseria, G. E.; Ernzerhof, M. hybrid functionals based on a screened coulomb potential. *J. Chem. Phys.* **2003**, *118*, 8207–8215.

(6) Perdew, J. P.; Burke, K.; Ernzerhof, M. generalized gradient approximation made simple. *Phys. Rev. Lett.* **1996**, *77*, 3865.

(7) Grimme, S.; Antony, J.; Ehrlich, S.; Krieg, H. a consistent and accurate ab initio parametrization of density functional dispersion correction (dft-d) for the 94 elements h-pu. *J. Chem. Phys.* **2010**, *132*, 154104.

(8) Gali, A.; Janzén, E.; Deák, P.; Kresse, G.; Kaxiras, E. theory of spin-conserving excitation of the $n - v^{-}$ center in diamond. *Phys. Rev. Lett.* **2009**, *103*, 186404.

(9) Freysoldt, C.; Neugebauer, J. first-principles calculations for charged defects at surfaces, interfaces, and two-dimensional materials in the presence of electric fields. *Phys. Rev. B* **2018**, *97*, 205425.

(10) Christiansen, O.; Koch, H.; Jørgensen, P. the second-order approximate coupled cluster singles and doubles model cc2. *Chem. Phys. Lett.* **1995**, *243*, 409–418.

(11) Schirmer, J. beyond the random-phase approximation: a new approximation scheme for the polarization propagator. *Phys. Rev. A* **1982**, *26*, 2395.

(12) Ahlrichs, R.; Bär, M.; Häser, M.; Horn, H.; Kölmel, C. electronic structure calculations on workstation computers: the program system turbomole. *Chem. Phys. Lett.* **1989**, *162*, 165–169.

(13) TURBOMOLE V6.4 2017, a development of university of karlsruhe and forschungszentrum karlsruhe gmbh, 1989-2007, turbomole gmbh, since 2007.

(14) Angeli, C.; Cimiraglia, R.; Evangelisti, S.; Leininger, T.; Malrieu, J.-P. introduction of n-electron valence states for multireference perturbation theory. *J. Chem. Phys.* **2001**, *114*, 10252–10264.

(15) Neese, F. software update: the orca program system, version 4.0. *Wiley Interdiscip. Rev. Comput. Mol. Sci.* **2018**, *8*, e1327.

(16) Dunning Jr, T. H. gaussian basis sets for use in correlated molecular calculations. i. the atoms boron through neon and hydrogen. *J. Chem. Phys.* **1989**, *90*, 1007–1023.

(17) Perdew, J. P.; Ernzerhof, M.; Burke, K. rationale for mixing exact exchange with density functional approximations. *J. Chem. Phys.* **1996**, *105*, 9982–9985.

(18) Giannozzi, P.; Baroni, S.; Bonini, N.; Calandra, M.; Car, R.; Cavazzoni, C.; Ceresoli, D.; Chiarotti, G. L.; Cococcioni, M.; Dabo, I. et al. quantum espresso: a modular and open-source software project for quantum simulations of materials. *J. Phys. Condens. Matter* **2009**, *21*, 395502.

(19) Schlipf, M.; Gygi, F. optimization algorithm for the generation of oncv pseudopotentials. *Comput. Phys. Commun.* **2015**, *196*, 36–44.

(20) Ge, X.; Binnie, S. J.; Rocca, D.; Gebauer, R.; Baroni, S. turbotddft 2.0 hybrid functionals and new algorithms within time-dependent density-functional perturbation theory. *Comput. Phys. Commun.* **2014**, *185*, 2080–2089.

(21) Sohier, T.; Calandra, M.; Mauri, F. density functional perturbation theory for gated two-dimensional heterostructures: theoretical developments and application to flexural phonons in graphene. *Phys. Rev. B* **2017**, *96*, 075448.

(22) Thiering, G.; Gali, A. the $(e_g \bigotimes e_u) \bigotimes E_g$ product jahn-teller effect in the neutral group-iv vacancy quantum bits in diamond. *Npj Comput. Mater.* **2019**, *5*, 18.

(23) Ciccarino, C. J.; Flick, J.; Harris, I. B.; Trusheim, M. E.; Englund, D. R.; Narang, P. strong spin-orbit quenching via the product jahn-teller effect in neutral group iv qubits in diamond. *npj Quantum Mater.* **2020**, *5*, 75.

(24) Pershin, A.; Hall, D.; Lemaur, V.; Sancho-Garcia, J.-C.; Muccioli, L.; Zysman-Colman, E.; Beljonne, D.; Olivier, Y. highly emissive excitons with reduced exchange energy in thermally activated delayed fluorescent molecules. *Nat. Commun.* **2019**, *10*, 1–5.

(25) Bersuker, I. *the jahn-teller effect*; Cambridge University Press, 2006.

(26) Goldman, M. L.; Doherty, M.; Sipahigil, A.; Yao, N. Y.; Bennett, S.; Manson, N.; Kubanek, A.; Lukin, M. D. state-selective intersystem crossing in nitrogen-vacancy centers. *Phys. Rev. B* **2015**, *91*, 165201.

(27) Wu, F.; Smart, T. J.; Xu, J.; Ping, Y. carrier recombination mechanism at defects in wide band gap two-dimensional materials from first principles. *Phys. Rev. B* **2019**, *100*, 081407.

(28) Smart, T. J.; Li, K.; Xu, J.; Ping, Y. intersystem crossing and exciton–defect coupling of spin defects in hexagonal boron nitride. *Npj Comput. Mater.* **2021**, *7*, 59.

(29) Brawand, N. P.; Vörös, M.; Galli, G. surface dangling bonds are a cause of b-type blinking in si nanoparticles. *Nanoscale* **2015**, *7*, 3737–3744.

(30) Hayee, F.; Yu, L.; Zhang, J. L.; Ciccarino, C. J.; Nguyen, M.; Marshall, A. F.; Aharonovich, I.; Vučković, J.; Narang, P.; Heinz, T. F. et al. Revealing multiple classes of stable quantum emitters in hexagonal boron nitride with correlated optical and electron microscopy. *Nat. Mater.* **2020**, *19*, 534–539.
